# Ontology-based dietary recommendation system for Chinese children and adolescents: development and a pilot validation study

**DOI:** 10.3389/fpubh.2026.1780898

**Published:** 2026-05-22

**Authors:** Zhiyu Zhang, Dongdong Xu, Jiye An, Min Yang, Sasa Xie, Ning Deng

**Affiliations:** 1 College of Biomedical Engineering and Instrument Science, Ministry of Education Key Laboratory of Biomedical Engineering, Zhejiang University, Hangzhou, China; 2 Department of Nutrition and Food Hygiene, School of Public Health, Zhejiang University School of Medicine, Hangzhou, China; 3 Center of Clinical Big Data and Analytics, The Second Affiliated Hospital, Zhejiang University School of Medicine, Hangzhou, China; 4 Binjiang Institute, Zhejiang University, Hangzhou, China

**Keywords:** artificial intelligence, children and adolescents, dietary recommendation, multi-criteria decision-making, ontology

## Abstract

**Background:**

Dietary imbalances among Chinese children and adolescents are prevalent, but most recommender systems are adult-oriented and not adapted to Chinese dietary patterns or school–family meal contexts. This study aims to developed and evaluated an ontology-based dietary recommendation system for Chinese children and adolescents to improve their dietary status.

**Methods:**

An ontology knowledge base was constructed from dietary guidelines, expert knowledge, and food and meal data. A multi-criteria decision framework generated feasible meal plans, optimized portion sizes with a genetic algorithm, and selected solutions that promote food diversity. We conducted a preliminary evaluation of the system in two pilot settings: (1) a group-level experiment using 30 days of school lunch data from a primary school, and (2) an individual-level experiment using 30 days of dietary records and health check-up data from 30 middle school students. Diet quality was assessed using the Chinese Children Dietary Index (CCDI), nutrient adequacy rates, and food-group diversity.

**Results:**

In the group experiment, system-recommended lunches achieved a significantly higher average CCDI score (117.39 ± 6.61) than actual school lunches (103.41 ± 9.44, *p* < 0.001). In the individual experiment, recommended full-day meals scored higher in CCDI (110.91 ± 9.70) compared with self-selected diets (90.61 ± 10.77, *p* < 0.001). Across both settings, the system reduced deficiencies in vegetables, fruits, and aquatic products with consistent improvements in nutrient adequacy and food diversity, while maintaining alignment with dietary guidelines.

**Conclusion:**

To the best of our knowledge, this study presents the first ontology-driven dietary recommendation system tailored for Chinese children and adolescents. By integrating structured knowledge representation with advanced decision-making algorithms, the system demonstrates promising improvements in dietary quality, nutritional balance, and food diversity across both school and family scenarios. These findings highlight the system’s potential as a practical tool for promoting healthy eating behaviors, and informing nutrition education.

## 1 Introduction

Diet and health are intricately interlinked. A balanced diet supplies the body with vital energy and sufficient nutrients necessary for sustaining normal physiological functions, while also mitigating the risk of chronic conditions, including cardiovascular diseases, diabetes, and obesity (1–3). Particularly for children and adolescents undergoing critical growth and developmental phases, a healthy and balanced diet is indispensable. It not only facilitates the realization of their full growth potential (4) but also establishes a robust foundation for cultivating healthy eating behaviors into adulthood (5). Nevertheless, the prevailing dietary and nutritional habits of children and adolescents are worrisome, marked by excessive caloric intake coupled with inadequate nutrients, insufficient consumption of fruits and vegetables, and an over-consumption of salt and sugar (6, 7). The Report on Nutrition and Chronic Diseases of Chinese Residents (2020) reveals that the stunting prevalence among children and adolescents aged 6 to 17 is 2.2%, the anemia rate is 6.1%, and the overweight and obesity prevalence rates are 11.1 and 7.9%, respectively (8). Consequently, concerted efforts are imperative to enhance the dietary patterns of children and adolescents, ensuring they receive adequate and balanced nutritional intake.

Enhancing the dietary nutrition status of children and adolescents necessitates a comprehensive understanding of their population characteristics and daily eating patterns (9). Primarily, children and adolescents experience rapid growth and development, with evolving physiological and nutritional needs that vary with age. Common health concerns, including obesity and anemia, can be directly or indirectly mitigated by optimizing dietary nutrient content and food composition (10–12). Consequently, addressing adolescent dietary health requires a focus on multiple factors, including nutritional balance and food diversity, to develop dietary strategies that adhere to established guidelines. Secondly, unlike Western diets, Chinese diets are based on grains and cereals with moderate amounts of vegetables and meat, emphasizing a balance between meat and vegetables, the integration of diverse ingredients (13, 14), and attention to color, aroma, and flavor, frequently enhanced by a wide range of seasonings. For children and adolescents, prolonged consumption of traditional Chinese diets without professional guidance or appropriate pairing may result in issues such as excessive salt, oil, and sugar intake (15). Consequently, effective dietary guidance for Chinese children and adolescents requires an in-depth understanding of the structure and characteristics of Chinese cuisine, aiming to address dietary health issues by appropriately matching dishes and portions while preserving traditional eating habits.

In addition, adolescents’ dining scenarios are diverse, with schools and homes serving as their primary meal settings (16), each characterized by distinct methods of meal preparation and provision. Family meal settings are typically prepared by parents or grandparents, influenced by family economic conditions, dietary concepts, and cultural traditions, with certain intergenerational differences (17); whereas school meal settings are more influenced by policy regulation and collective meal arrangements, characterized by standardization and bulk provision (18). Compared with meal settings for children and adolescents in other countries or regions, Chinese settings display a distinct collective nature and family involvement. For example, in Europe and the United States, adolescents tend to develop the ability to independently choose their meals at an earlier age and often solve part of their meals through convenience foods, demonstrating stronger individual autonomy and convenience orientation (19, 20). In contrast, in China, families have stronger dietary interventions for adolescents (21), and schools also play an important role in nutritional intervention. Additionally, compared with adults’ diets, children’s and adolescents’ eating behaviors are more susceptible to external influences. Due to their immature cognitive level, dietary self-control, and the formation of dietary preferences, they are more easily affected by advertisements, peers, and family dietary culture, leading to tendencies toward high sugar, high salt, and unbalanced eating habits (22, 23). Adults, on the other hand, have greater dietary freedom and consumer choice, their eating habits are more stable, and they are more likely to be driven by self-managed nutrition motivation or chronic disease management needs. Thus, achieving a balanced diet for children and adolescents necessitates tailoring dietary recommendations to their dining scenarios, emphasizing guidance on eating behaviors and environmental adaptability and crafting personalized meal plans.

## 2 Related work

Currently, there are various systems in the field of dietary recommendation, with different focuses, application scenarios, and technical architectures. Yet systematic reviews reveal that the field tends to be skewed toward adult users and Western dietary contexts. A recent systematic review of 67 food recommender systems found that most systems are built using content-based filtering approaches with data predominantly sourced from Western recipe databases, and that several studies ignore personal attributes of users when producing recommendations (24). Similarly, a comprehensive review of health recommender systems noted that nutrition-related recommendations constitute a significant category, but the vast majority target general adult populations, with children and adolescents being markedly underrepresented as end users (25). These findings underscore a critical gap: the absence of food recommender systems designed to accommodate the distinctive nutritional needs, dining contexts, and dietary cultures of children and adolescents, particularly in non-Western settings. Representative systems in the field illustrate these limitations concretely. PREFer by Bianchini et al. (26) takes into account food preferences and medical prescriptions to provide users with personalized, healthy meal lists; SousChef by Ribeiro et al. (27) is aimed at the older population and generates personalized meal lists based on body measurements, preferences, and activity levels; and Stefanidis et al. (28)’s PROTEIN AI Advisor combines an expert-validated database with a two-tier architecture to create daily or weekly meal plans for healthy and health-status-specific populations. Despite their technical merit, these systems share three fundamental limitations that preclude their direct application to Chinese children and adolescents. First, they model meals as single-item recommendations or simple item lists, which cannot capture the combinatorial structure of Chinese meals comprising multiple dish types with inter-dish constraints on ingredient diversity and nutritional complementarity. Second, they lack pediatric nutritional modeling that incorporates age- and growth-stage-specific nutrient targets or health-condition-aware adjustments. Third, they do not account for the dual school–family dining context that characterizes Chinese children’s meal environments, where school canteens follow group-level standardized provision while family meals are individually customized.

To build an intelligent dietary recommendation system for children and adolescents first requires an authoritative and comprehensive knowledge base as a decision support. Ontology, as a formalized shared conceptual model, can express object types, attributes and their relationships in a structured and semantic way, enabling the system to process complex information more accurately and flexibly (29–31). Given the structural complexity and nutritional reasoning needs of Chinese diets, ontology is the preferred solution for constructing this type of knowledge base. Existing dietary knowledge bases and ontology studies include Food Ontology (FoodOn) (32), Food Knowledge Graph (FoodKG) (33), Ontology for Nutritional Studies (ONS) (34), etc., all of which achieve unified food terminology, structured nutritional knowledge and support computational reasoning. However, these ontologies are structurally insufficient for dietary recommendation in our target population. First, these ontologies are designed around Western food taxonomies organized at the individual food-item or ingredient level, and lack the hierarchical modeling capacity needed to represent Chinese multi-dish meal structures, where a single meal comprises coordinated combinations of dishes, each composed of multiple ingredients. Second, they do not encode age-stratified nutritional targets or growth-stage-specific dietary requirements; the Dietary Reference Intakes for children and adolescents vary substantially across age groups, yet existing ontologies treat nutritional standards as static adult-oriented values. In addition, these ontologies lack the inferential capacity to reason about health-condition-specific dietary adjustments, which requires not only food–nutrient mappings but also user–health–condition–diet linkage rules. These gaps necessitate the development of a domain-specific, executable ontology that can model population characteristics, dining scenarios, and the complex structure of Chinese cuisine simultaneously.

Secondly, to build an intelligent dietary recommendation system for children and adolescents requires the consideration of demographic and contextual characteristics to address diverse nutritional needs. Thus, the method of multi-criteria decision-making (MCDM) can be introduced, which is a kind of decision-making method to deal with multi-objective conflicts by integrating group opinions, quantifying weights, and using analytical techniques to optimize solution selection (35). It is mainly divided into multi-attribute decision-making (MADM) and multi-objective decision-making (MODM), which can provide more personalized and balanced recommendations for children’s and adolescents’ nutrition. Among the available studies, Gazan et al. (36) summarized the process of applying mathematical optimization methods to develop dietary plans; Salloum et al. (37) used genetic algorithms and particle swarm optimization to create personalized meal plans and compared them with linear programming; Gaál et al. (38) proposed multilevel genetic algorithms to simultaneously satisfy the constraints of nutrition, preferences, and food compatibility. These existing optimization approaches predominantly operate at the food-group or ingredient level, rather than at the dish level that characterizes real meal provision in Chinese dining contexts. This abstraction gap means their outputs cannot be directly translated into actionable, dish-based meal plans without an additional, non-trivial composition step. Moreover, most studies focus exclusively on nutritional adequacy as the optimization objective, treating food diversity as at best a secondary constraint rather than an equally important optimization target. For children and adolescents, however, food diversity is not merely a preference but a nutritional imperative linked to micronutrient adequacy and the development of healthy eating behaviors. Our study addresses these gaps by proposing a unified framework that operates at the dish level, treats food diversity as a co-equal optimization criterion, and integrates MODM and MADM in a coherent two-phase pipeline.

## 3 Methods

### 3.1 Study overview

To address the lack of an executable, China-specific knowledge base and algorithmic framework for healthy eating among children and adolescents, this study aims to (1) construct a domain ontology that formalizes demographic characteristics, dining scenarios, foods, and nutrient targets, to adapt to specific populations and scenarios; (2) design a dietary recommendation algorithm that integrates rule-guided meal composition with multi-objective portion optimization and diversity-aware solution selection, to simultaneously satisfy nutritional balance and food diversity; and (3) implement a multi-scenario system, to support both school meal services and family dining. We evaluate effectiveness against real-world baselines using quantifiable endpoints: overall diet quality, nutritional balance, and food diversity. By achieving these outcomes, the study seeks to improve children’s and adolescents’ dietary quality and enable scalable adoption in both institutional and home settings. We did not include head-to-head comparisons with established optimization-based or recommender-system baselines in this study, because most existing approaches operate at the food-group or ingredient level and are not directly compatible with our dish-level, scenario-specific recommendation framework.

**Figure 1:**
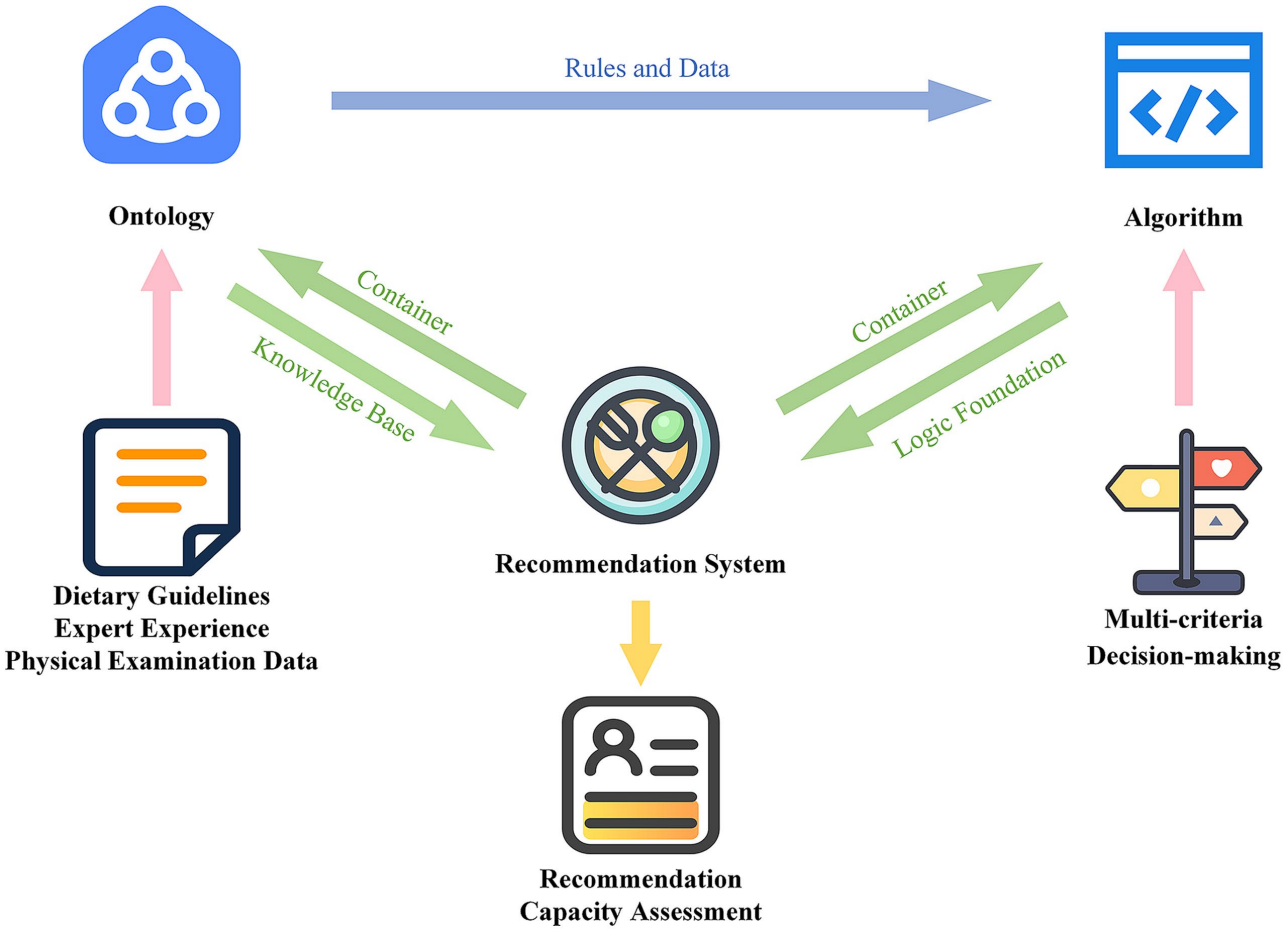
Overall study design.

The study was designed based on these tasks. Figure 1 illustrates the overall study design. The basis of the recommendation process is a customized ontology called the Child and Adolescent Dietary Recommendation Ontology (CADRO), which consists of dietary guidelines, expert insights, and additional relevant information, and is used to provide rules and data for the recommendation algorithm. The core of the recommendation process is the Child and Adolescent Dietary Recommendation Algorithm (CADRA), which leverages the ontology’s knowledge and employs a multi-criteria decision-making framework to generate personalized recommendation meals for users. Finally, the implementation of the recommendation process is facilitated by the developed Child and Adolescent Dietary Recommendation System (CADRS), which offers users a terminal through which the application of the ontology and algorithms can be executed to evaluate recommendation efficacy.

### 3.2 Ontology construction

Ontology is a structured approach for organizing and representing knowledge, which helps systems better understand and process complex information by explicitly defining concepts and their relationships within a domain (39). According to the degree of domain dependency, ontologies can be categorized into four types: top-level ontologies, domain ontologies, task ontologies, and application ontologies (40). In Chinese dietary patterns, dishes exhibit diverse structures, flexible combinations, and complex rules, so a knowledge representation method that supports semantic reasoning and interpretability is needed to effectively model the intricate logic among different ingredients, dishes, and nutritional components. Compared with traditional rule tables or relational databases, ontologies have significant advantages in expressing multi-level nutritional knowledge, handling uncertainty and providing interpretable recommendations. Based on this consideration, this study constructed the Child and Adolescent Dietary Recommendation Ontology (CADRO), an application ontology specifically geared toward personalized dietary recommendation scenarios for children and adolescents. The development of this ontology followed the seven-step approach proposed by Stanford University (41) and was completed by this research team in collaboration with nutrition experts. Since CADRO involves sensitive physical examination records and unpublished dietary data, the full ontology populated with instances is not hosted in a public repository to protect privacy. However, the ontology structure and related resources are available for research purposes upon request (see Data Availability Statement).

CADRO’s knowledge comes from two main sources: manual collection and processing, and rule-based reasoning generation. The standard food instances included nearly 5,000 entries provided by the Chinese Food Composition Table (42), with attributes and relationships either directly sourced from the table or verified and refined by nutrition experts. Knowledge related to health issues was derived from various guidelines and books on children’s nutrition to extract dietary recommendations for child and adolescent health issues, which were subsequently converted into relational descriptions between corresponding instances. These sources included the Dietary Care Guidelines for Growth-Stunted Children and Adolescents (43), the Guidelines for Medical Nutrition Treatment of Overweight/Obesity in China (44), and the Chinese Hypertension Prevention and Treatment Guidelines (45).

Considering the diversity of dietary health groups among children and adolescents and the uniqueness of Chinese diets, the knowledge was structured into three primary modules, namely user profiling, dietary health, and dietary goals, and relevant concepts and attributes were systematically summarized for each module. In the user profiling module, two-dimensional features, including basic characteristics and health issues, were summarized and processed based on student physical examination data and expert experience. Six concepts under basic characteristics were defined as its data attributes, and five concepts under health issues were categorized as third-level concepts within the ontology. In the dietary health module, dietary guidelines were organized, field investigations were conducted, and expert advice was gathered to identify eight primary concepts (e.g., food, ingredients, and dishes), six attribute-related concepts, and multiple secondary and tertiary concepts. In the dietary goals module, elements such as set meals, nutrients, dietary pagoda food groups, and food were organized within the dietary health module, while other entity concepts were defined as classes in the ontology. Ultimately, four top-level concepts and 27 subordinate concepts were derived through reasoning. Some of the key concepts of dietary health can be found in the Supplementary File S1.

CADRO was modeled using Protégé software and semantically expressed in the OWL language. Class and attribute naming followed the English CamelCase rules with Chinese labels, identifiers were automatically assigned by Protégé, and some entities reused the standardized descriptions of FoodOn (32), ONS (34), and SNOMED CT (46) to maintain semantic consistency and interoperability. In the ontology relationship design, the core relationships such as containment, correspondence, and concern were defined, and the OWL axiomatic schema was used to express the hierarchical inheritance and constraints.

### 3.3 Algorithm design

Dietary recommendations for children and adolescents are highly concerned about nutritional health quality and focus on professionalism and interpretability, and we use a rule-based approach allowing for the integration of expertise to optimize recommendations and can compensate for the shortcomings of the cold-start problem (47). There is a multi-criteria decision-making (MCDM) (33) problem in the process of dietary recommendations, and the rational analysis and adoption of MCDM can help provide more personalized, comprehensive and balanced dietary recommendation suggestions.

CADRA is divided into three steps, each supported by the dietary recommendation knowledge base. According to the analysis of the meal recommendation process for children and adolescents, there are both a multi-objective decision-making (MODM) problem of optimizing from an infinite set of alternatives, and a multi-attribute decision-making (MADM) problem of sorting from a finite number of alternatives. Therefore, the algorithm adopts an integrated MCDM strategy. In this approach, the system first applies MODM to generate a Pareto-optimal set of balanced meal plans under nutritional adequacy and energy sufficiency. Then, MADM methods are used to rank and select the final recommendations that most closely matches food diversity needs. This two-phase “optimization–selection” paradigm has been validated in MCDM and multi-objective optimization research (48) and has demonstrated strong effectiveness in healthcare and food-engineering contexts (49–51).

#### 3.3.1 Dish combination

For the different dining scenarios of children and adolescents, we formulated the rules for dish combinations based on the Dietary Guidelines for Chinese Residents (52), while also referring to the 30-day actual meal supply data of an elementary school in Pinghu City. Breakfast consists of “1 staple food + 1 main meat dish + 1 soup,” while lunch and dinner are designed with three types of dish combinations: 1 staple food + 1 main meat dish + 1 semi-meat dish + 1 vegetarian dish, 1 staple food + 1 main meat dish + 1 semi-meat dish + 2 vegetarian dishes, and 1 staple food + 1 main meat dish + 1 vegetarian dish. Soup is not a mandatory component, and the staple food may include two dishes. In addition to the rules for the combination of dish types, there are other constraints for the pairing of single meals as well as one-day and one-week set meals.

To implement the dish combination rules, corresponding algorithmic processes are developed for single set meals, one-day set meals, and one-week set meals. The input data for CADRA includes the user’s personalized meal goals, a comprehensive meal library with attributes, and user-defined, partially configurable meal requirements, encompassing meal composition types and meal time. The following describes in detail the combination process for single, 1-day, and 1-week set meals, and the combination rules can be found in Supplementary File S2.

In the single set meal combination process, the algorithm first randomly selects a form of staple food, and then randomly picks a dish from the corresponding category. For example, when choosing “fine grain staple food + coarse grain staple food,” one could get “white rice + steamed sweet potato.” For vegetarian dishes, if the set meal requires two vegetarian dishes, one vegetarian dish is randomly selected first, and then another different dish is chosen based on its main ingredients to ensure variety., e.g., “okra with garlic + green beans and tofu.” Similarly, for meat dishes, the main meat dish is selected first, and then according to the main ingredient category, the semi-meat dish of different food categories is filtered out, like “braised pork chop + stir-fried beef with bamboo shoots.” Through this process, the final combination of staple food, meat and vegetarian dishes for the meal is obtained.

The one-day set meal consists of a combination of three meals. Breakfast has fewer rules and is therefore realized separately. Lunch and dinner are subject to additional requirements, e.g., at least one meal contains a roughage staple and at least one meal contains dark vegetables. The remaining unduplicated requirements are realized by reducing the candidate meal options and selecting them randomly. If the first round of dish combination does not meet the food quantity requirements, the algorithm will proceed with a second round, prioritizing meals with more food items.

The one-week set meal combination process mainly constrains the number of intake of foods such as aquatic products, soy products, and mushrooms and algae. The algorithm first breaks down the food quantity requirements for 7 days according to the dietary guidelines, and then generates a combination plan for each day based on the 1-day set meal pairing process. In particular, the special food categories correspond to specific dish types: aquatic products are included in main meat meals, soybean products are included in vegetarian or semi-vegetarian meals, and mushrooms and algae are featured in vegetarian meals.

#### 3.3.2 Servings calculation

After completing the dish combination, the algorithm uses multi-objective optimization to calculate the weight of each meal in order to satisfy the nutrient intake requirements specified in the user’s dietary goals. In this problem model, in addition to satisfying the recommended intake of different nutrients, there should also be weight range constraints for each dish to prevent the solution of unreasonable or infeasible weights. The user’s personalized dietary goals specify reference intake quantities for each food group in the dietary pagoda. These are then converted into weight constraint ranges for different dish types. For instance, if a user’s reference intake of cereals and potatoes is 140–160 grams, the corresponding weight constraints for staple foods may be set between 100–200 grams. This range is moderately expanded to prevent imposing excessive constraints on the genetic algorithm’s calculations and to accommodate the flexibility of actual meal preparation.

The mathematical model is illustrated through the calculation of servings in a single set meal. The combination of dishes is defined as staple food $X_{0}$ + main meat dish $X_{1}$ + semi-meat dish $X_{2}$ + vegetarian dish $X_{3}$ . The nutrient range provided in this meal is determined by the selected meal time and age group, expressed as $lu^{j},hu^{j}$ , where j denotes the nutrient type index for fourteen nutrients, including energy, protein, fat, carbohydrate, dietary fiber, vitamin A, vitamin B1, vitamin B2, vitamin C, iron, sodium, calcium, zinc, and potassium. The weight range for each dish type in this meal is defined as $\left[lw^{i},hw^{i}\right]$ , based on the selected meal time and age group, where denotes the dish type index, which can take values of 0, 1, 2, or 3.

Let the raw weight of dish $X_{i}$ , i.e., standard weight, be $ow_{i}$ , the actual weight in the set meal is also $nw_{i}$ . Here, $nw_{i}$ serves as the independent variable of the optimization model, while the nutrient content provided by each dish is represented as $u_{i}^{j}$ . Consequently, the total content of each nutrient in the entire set meal can be calculated using Equation (1):

$$au^{j}=\sum_{i=0}^{4}\frac{nw_{i}\times u_{i}^{j}}{ow_{i}}$$ (1)

For certain nutrients, their content will be constrained not only by nutrient ranges but also by health labels, requiring their intake to meet a specific value, as defined in Equation (2).

$$au^{q}=\sum_{i=0}^{3}\frac{nw_{i}\times u_{i}^{q}}{ow_{i}}=H,$$ (2)

where q denotes the index of the particular nutrient, and H represents the prescribed intake for that nutrient. For instance, if the user is anemic, an increased intake of iron is required (53). To ensure that each nutrient provided in the meal remains within the recommended range, i.e., $lu^{j}<au^{j}<hu^{j}$ , 14 objective functions can be established to represent each of the 14 nutrients under consideration, as shown in Equation (3):

$$f{\left(\mathrm{nw}\right)}^{j}=\frac{|au^{j}–lu^{j}|+|au^{j}–hu^{j}|–(hu^{j}–lu^{j}|)}{hu^{j}},j=0,1,2,\ldots 13$$ (3)

It can be observed that when the numerator of an objective function is zero, i.e., when the entire objective function value equals zero, the meal supplies that nutrient within the recommended range. Conversely, a larger value of the objective function signifies that the meal supplies that nutrient further away from the recommended range.

In summary, the mathematical model for nutritional calculations includes an independent variable, $\;nw_{i}$ , and a total of 14 objective functions aimed at minimizing the values of these functions to zero. Furthermore, the independent variables are constrained by the weight ranges of each dish type in a meal, while specific nutrients are regulated by health labels. The overall computational model is illustrated in Equation (4):

$$\left\{ \begin{array}{c} f{\left(\mathrm{nw}\right)}^{j}=\frac{|au^{j}–lu^{j}|+|au^{j}–hu^{j}|–(hu^{j}-lu^{j})}{hu^{j}},j=0,1,2,\ldots 13,\\ au^{q}=H,q\in \{0,1,2\ldots 13\},\\ lw^{i}\leq nw^{i}\leq hw^{i},i=0,1,2,3. \end{array}\right.$$ (4)

Given that a large number of objectives may increase computational complexity and decrease population diversity, thereby affecting the accuracy of results, we merged the relevant nutrients during the actual solving process, ultimately reducing the 14 objective functions to 7.

We used the decomposition-based multi-objective evolutionary algorithm (MOEA/D) to solve this optimization problem. Compared with non-dominated sorting algorithms such as NSGA-II and SPEA2, MOEA/D decomposes a multi-objective problem into several scalar subproblems and evolves them collaboratively. This approach shows superior performance in high-dimensional and constrained domains with lower computational complexity and balanced convergence–diversity (54–56). Specifically, we implemented the optimization algorithm using the MOEA Framework (57), an open-source Java library for multi-objective optimization. The Tchebycheff aggregation function was used as the decomposition method. The initial population size was automatically determined by the weight vector generator, ensuring that the number of subproblems matches the number of weight vectors. The neighborhood size was set to 20, the mating selection probability was set to 0.9, and the maximum replacement count was set to 2. The stopping criterion was set to a maximum of 10,000 function evaluations, a value determined through preliminary convergence testing. Utilizing this algorithm, a non-dominated set of Pareto-optimal solutions can be derived, where the weights of the corresponding dishes meet the recommended range of nutrients in the meal plan to the greatest extent possible.

#### 3.3.3 Meal program generation

Due to the characteristics of multi-objective optimization problems, finding a solution that optimally satisfies all objectives is challenging (58). Therefore, after nutritional calculations, several feasible solutions can be obtained, each representing a potential distribution of dish portion sizes in the meal composition. To select one solution as the output, a distance-based decision-making approach is employed (59). From a nutritional perspective, dietary goals encompass two key aspects: nutritional balance, which corresponds to the recommended intake of various nutrients, and food diversity, which corresponds to the recommended intake of dietary pagoda food groups. The feasible solution set for dish portions in the current meal is derived from the calculated nutritional balance results, which are assumed to satisfy the nutritional balance requirements. Decision-making is further guided by considerations of food diversity. Food diversity is represented by 10 dietary pagoda food group subclasses, such as grains, tubers, and vegetables. The algorithm selects the solution with the smallest Euclidean distance to the ideal solution as the final output solution by comparing the gap between the supply and ideal intake of each solution on 10 food groups.

With the above design, the algorithm is able to comprehensively consider the user’s personalized dietary goals, nutritional balance, and food diversity, generating dietary recommendation plans that meet the requirements under multiple constraints.

### 3.4 System development

Based on the demand analysis, the multi-scenario meal recommendation system for children and adolescents should consist of a server side, a user terminal for the school feeding scenario, and a user terminal for the family meal scenario. To enhance the user experience, the system adopts differentiated technical solutions for the characteristics of different scenarios. In the school scenario, the main users are cafeteria managers and teachers, who work in a fixed environment, mainly use computers, and need efficient data management and maintenance functions (60), so the terminal is implemented in the form of web pages. In the family scenario, the users are children and parents. Considering the high usage rate of mobile devices and the convenient demand of “use and go” (61), the terminal is implemented by WeChat Mini Program.

Although user terminals are designed for the two main dining scenarios of children and adolescents, their core functions revolve around applying knowledge bases and recommendation algorithms for dietary recommendations. Consequently, a server-side application is developed to provide services for both user terminals through distinct interfaces. To ensure a high degree of code reusability and decoupling, the server-side design adopts a classic layered architecture and introduces modularization and scalability in the knowledge base and inference mechanism (62). Among them, the dietary recommendation knowledge base is constructed with the ontology as the core, and the user profile, dietary health rules, food-nutrient mapping, etc. are modularized and managed so that they can be updated independently. The reasoning and execution of the recommendation rules rely on the rule engine that separates the business rules from the system code, ensuring the system maintains its flexibility and maintainability over long-term use. The server side of the system is built based on Spring Boot framework and provides interfaces to the outside world through RESTful APIs (62). MyBatis Plus serves as the persistence tool, with data stored in a MySQL database. Maven is employed for project construction and dependency management.

### 3.5 Recommendation capacity assessment

To evaluate the recommendation capability of the CADRS and verify the accuracy and feasibility of the recommended meal plans, this study analyzed and compared the meal plans from actual scenarios with those recommended by the system based on real data. Nutritional quality and healthfulness of the meals were assessed by constructing relevant evaluation indexes through the direct use of quantitative data, including nutrient content and other metrics. The primary meal scenarios for children and adolescents included school and family. The meal recommendation system was applied in both contexts; thus, this study conducted meal recommendation experiments from both group and individual perspectives. The group recommendation experiment assessed the nutritional quality and healthfulness of meals in school group scenarios, while the individual meal recommendation experiment evaluated personalized health in the context of family-based individual dining scenarios.

#### 3.5.1 Data collection

Meal data and physical examination data were provided by the corresponding school canteens and relevant persons in charge. The data were formatted as Excel tables, which could be directly written as a Java program to manipulate and organize, and carry out conceptual alignment, relational extraction and reasoning. To ensure data quality, a trained research assistant was assigned to perform primary data entry, and a licensed dietitian independently checked the entries against the original records and verified the consistency of mappings. The principles for selecting experimental data were completeness and availability, meaning that the samples must have continuous records with no obvious omissions. As the study did not involve any additional experimental interventions, no inclusion and exclusion criteria were set in the traditional sense. All data were used after obtaining consent from the school and parents of the students and were de-identified.

#### 3.5.2 Assessment indicators

To comprehensively assess whether the recommended plan achieves sufficient value for nutritional health compared to real data, the dietary quality was measured across three dimensions: overall dietary quality, nutritional balance, and food diversity, guided by documents such as the Dietary Guidelines for Chinese Residents (52). Specific indicators were established for each dimension to reflect its level. The detailed definitions of these three indicators can be found in Supplementary File S3.

#### 3.5.3 Group recommendation experiment

The group recommendation experiment collected 30 days of lunch supply data from the cafeteria of an elementary school in Pinghu City, Zhejiang Province, China, with the meal program designed by the responsible dietitian. To analyze the nutritional composition of this meal data and recommend a systematic meal plan based on actual dishes, the original meal data was transformed according to the knowledge base framework for children and adolescents’ meal recommendations. The cafeteria supply data included a total of 101 meal instances. Each meal ingredient was matched to specific food item, and the basic attributes of the meals were calculated and verified. In the group recommendation experiment, a 30-day lunch dietary recommendation was conducted for a group of children and adolescents with only age specified, based on the meal library of a certain elementary school cafeteria. The nutritional quality and healthfulness of the recommended lunches were compared with those of the actual lunches served, according to the assessment indicators.

#### 3.5.4 Individual recommendation experiment

The individual recommendation experiment collected physical examination data and 30 days of meal data from 30 students at a middle school in Hangzhou City, Zhejiang Province, China, with meal plans chosen independently by the students. Similarly, the original meal data was transformed according to the knowledge base framework for children and adolescents’ dietary recommendations. The dietary data from 30 students included a total of 502 meal instances. Each meal ingredient was matched with specific food item, and the basic attributes of the meals were calculated and verified. In the individual recommendation experiment, each of the 30 users received a 30-day full-day set meal recommendation based on their personalized dietary goals using the actual dish library. The nutritional quality and healthfulness of the recommended and self-selected set meals were compared for each user, and the results of these comparisons were comprehensively analyzed and evaluated.

#### 3.5.5 Sample size considerations

The sample sizes used in the two validation experiments were selected to support a pilot validation design. In the group-level experiment, 30 days of cafeteria lunch supply data were used to generate 30 paired daily comparisons between actual and system-recommended lunch plans, with 101 meal instances included in the meal database. In the individual-level experiment, 30 middle-school students each provided 30 days of dietary records and health examination data, yielding 502 meal instances and 30 paired user-level comparisons between self-selected and system-recommended full-day meal plans.

This sample size is consistent with commonly used guidance for pilot studies, in which the primary aims are to assess feasibility, calibrate parameters, evaluate data-processing procedures, and obtain preliminary estimates of effect magnitude. A frequently cited rule of thumb recommends approximately 12 participants or observations per group for pilot studies (63), while other methodological guidance suggests that pilot sample size should be determined according to the expected effect size and the objectives of the future main study (64). Therefore, the use of 30 paired observations in each experiment was considered adequate for preliminary validation and for detecting medium-to-large differences in dietary quality, while remaining feasible for a single-site school-based implementation. As a sensitivity check, we used the standard normal-approximation sample size formula for detecting a standardized paired mean difference (65),

$$n=\frac{{\left(Z_{1-\alpha /2}+Z_{1-\beta }\right)}^{2}}{d^{2}}$$

Where the effect size d is defined as the mean within-pair difference divided by the standard deviation of the within-pair differences. Under a two-sided α of 0.05 and power of 0.80 yield, $Z_{1-\alpha /2}=1.96$ and $Z_{1-\beta }=0.84$ . With 30 paired observations, the minimum detectable standardized paired effect is approximately 0.51. Thus, the current sample size provides reasonable sensitivity for detecting approximately medium or larger paired effects, according to conventional effect-size interpretation, which is consistent with the pilot nature of this validation study (66).

## 4 Results

### 4.1 System development results

#### 4.1.1 Ontology construction

The current version of CADRO includes 177 classes, 42 object properties, 15 data properties, and 31 enumerated values for properties, with 138 classes reused from existing ontologies.

**Figure 2:**
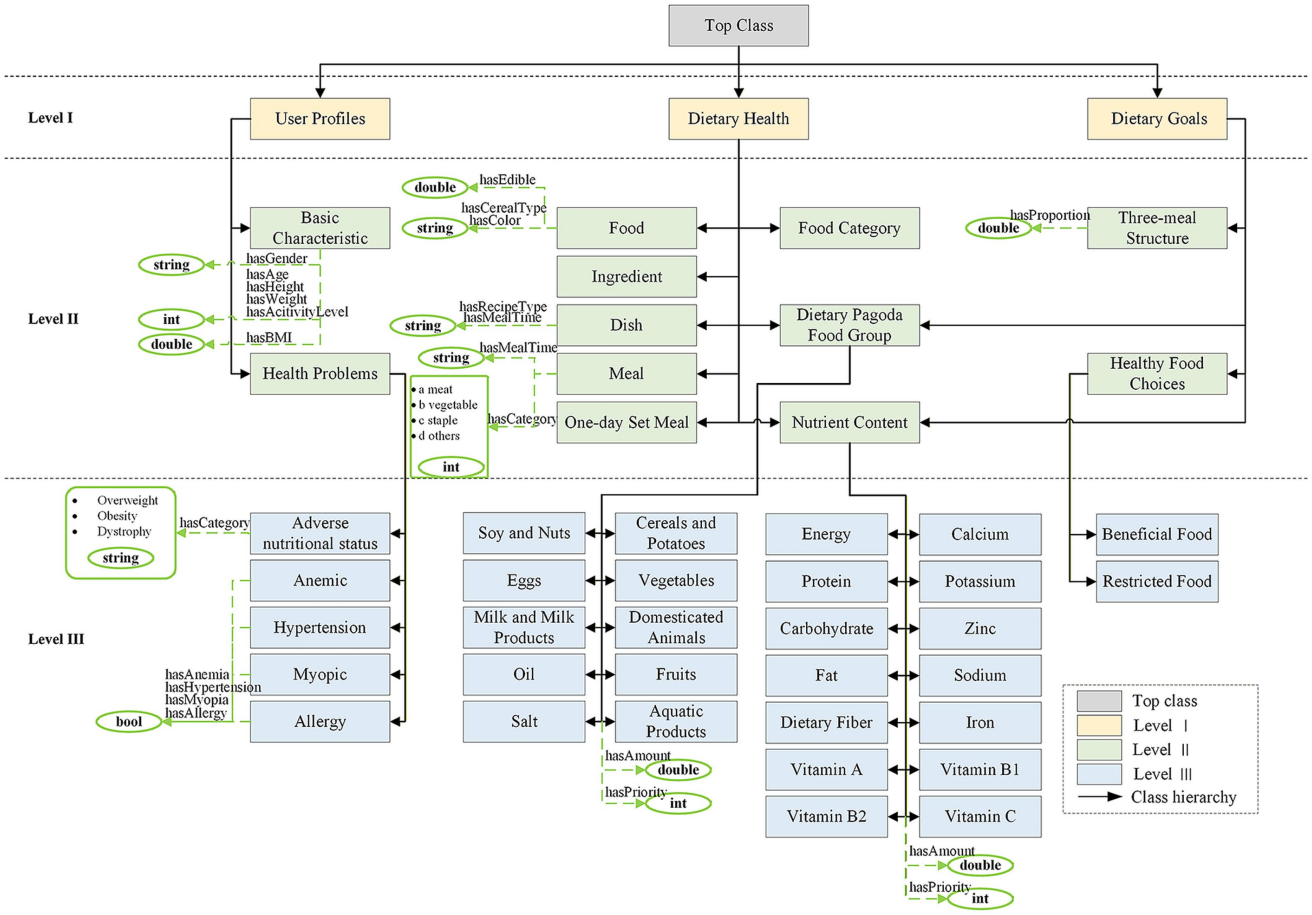
Class diagram of the Child and Adolescent Dietary Recommendation Ontology’s main core.

To ensure semantic correctness, the ontology was tested for logical consistency, and all classes and relations were verified to be conflict-free. Through this construction method, CADRO not only ensures transparency and reproducibility, but also has good scalability, providing a solid knowledge foundation for subsequent algorithm design and system integration. Figure 2 shows the main core class diagram of CADRO. We have not added all classes and attributes to the diagram for ease of presentation. For clarity, not all classes and attributes are depicted.

The instance layer of the constructed ontology closely integrates real data with the ontology. In terms of user data, the system reasoned personalized dietary goals based on information such as gender, age, height, weight, activity level, and health status. For example, it dynamically adjusts the priority of iron for users with anemia and generates suitable nutritional intake plans. In terms of meal data, based on the 30-day lunch supply data of an elementary school in Pinghu City, we applied deduplication, ingredient mapping, and rule-based reasoning to obtain each dish’s nutritional components and attributes such as dietary pagoda food group classification. This process ultimately produced a standardized meal database. The instance layer provides high-quality data that can be directly used by the recommendation algorithm.

#### 4.1.2 Algorithm design

**Figure 3:**
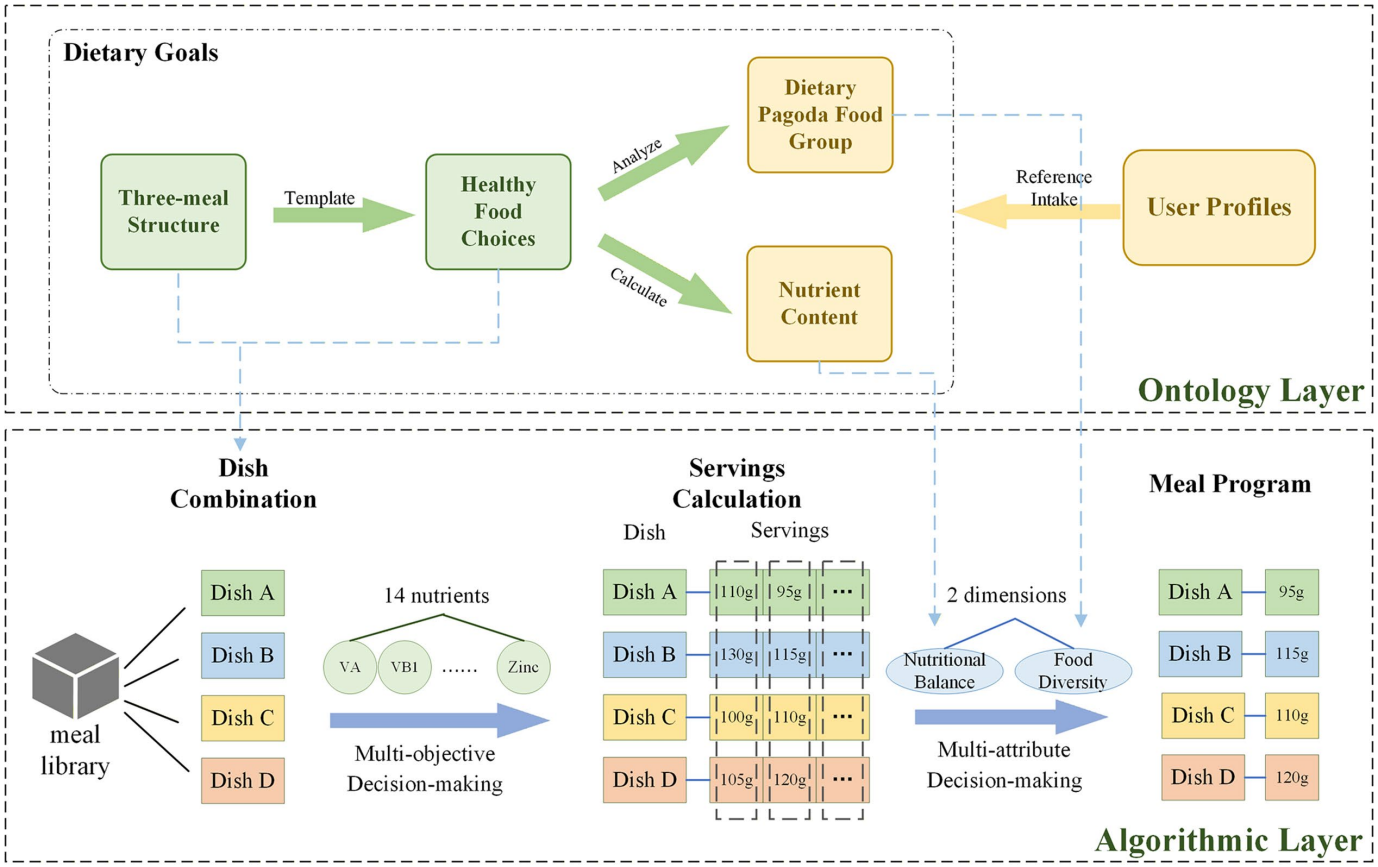
Process and framework for Child and Adolescent Dietary Recommendation Algorithm.

The algorithm design is divided into three main phases: dish combination, servings calculation and meal program as shown in Figure 3. The process begins with a rule-guided dish combination stage, where the system generates meal combinations that meet nutritional structure requirements, to reduce the occurrence of unreasonable combinations. Next, in the servings calculation stage, a multi-objective evolutionary algorithm is employed to optimize 14 nutritional targets, ensuring that the energy and micronutrient intake per meal meets established standards. In the final meal program stage, a food group distance-based method is used to prioritize solutions that are closer to the ideal diversity point, effectively preventing the overuse of single food ingredients.

The three design stages of the algorithm work in synergy, leading to improvements in the overall performance of the dietary recommendation system, particularly in terms of nutritional balance and food diversity.

#### 4.1.3 System development

In both terminals, users can realize the functions of editing user profiles and obtaining meal recommendations, etc. The web terminal also supports the addition, deletion, checking and modification of food and meal data for easier data management and maintenance, while the mini-program terminal supports the recording of three meal data, which is convenient for individual users to track their diets. All operation data is synchronously uploaded to the server for data persistence storage, and the core business logic processing is undertaken by the backend knowledge base and recommendation algorithm module.

**Figure 4:**
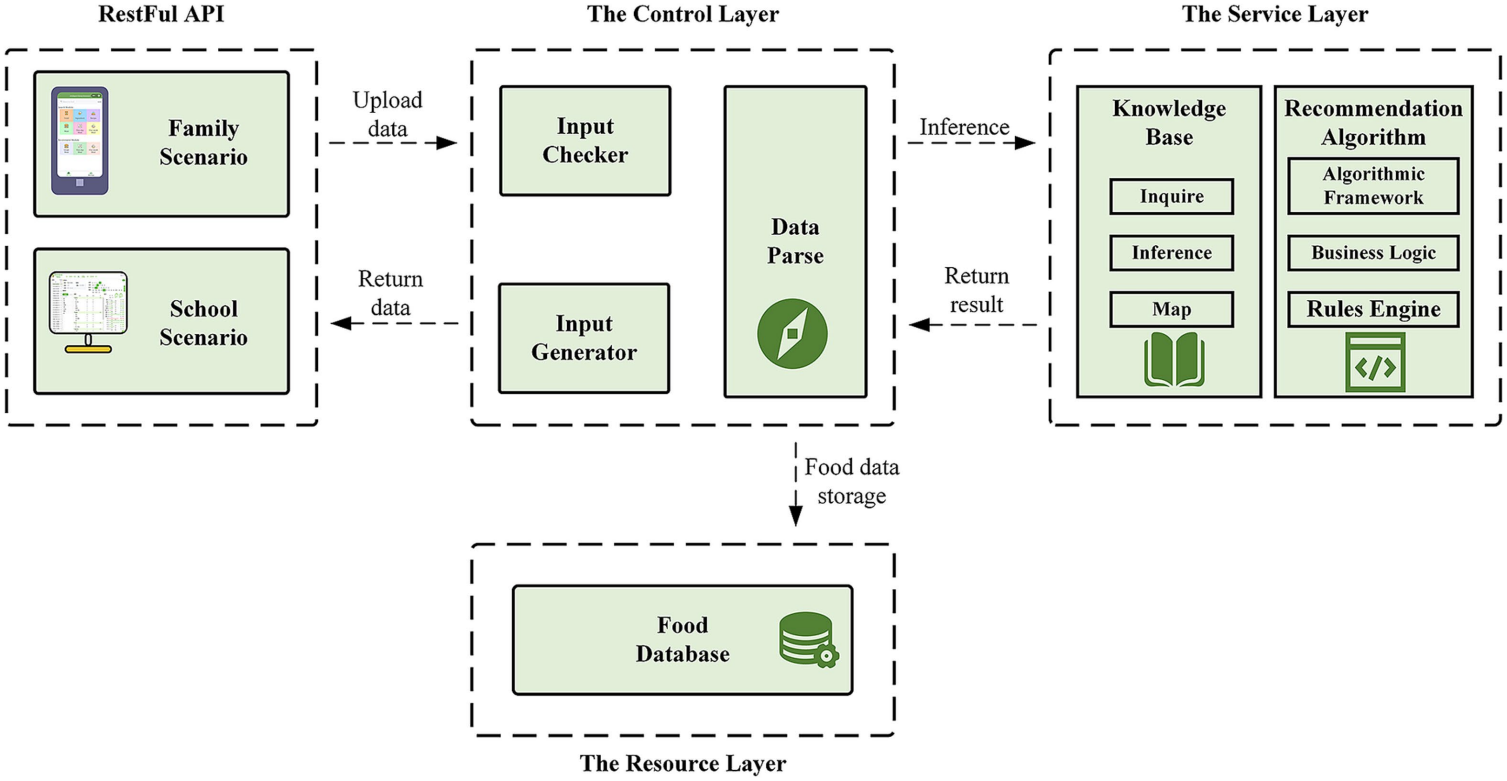
Structure of the system.

When the user terminal sends a request, it first requests a specific API corresponding to the interface layer. The request is intercepted by the control layer, responsible for preliminary input validation and data parsing, without directly handling business logic. If validation and parsing are successful, the request is passed to the service layer for core business logic processing. If the task involves complex recommendation rules or calculations, it will be processed by the knowledge base rules module and recommendation algorithm module. Next, the data is persistently stored, and operations such as adding, deleting, modifying, and checking are performed on the database. Finally, the results obtained from the service layer and the resource layer are sent back to the control layer in a layered manner, where they are encapsulated in JSON format for highly efficient data interaction by the user terminals. Throughout this process, all requests from the user terminal are executed by calling APIs. The core APIs provided by the server-side correspond to three primary functions of the meal recommendation process: meal target inference, meal collection management, and meal plan recommendation. Figure 4 illustrates the overall architecture of the system.

Ultimately, users can freely view the data and information of the food and meal databases on the web and mini-program platforms, set personalized requirements, and get meal recommendation results. For possible exceptions, such as dietary taboos or religious dietary restrictions, we have introduced a “configurable rules interface” in the system. Users can independently adjust the meal pairing rules in the system, replace or delete specific ingredients, or add personalized preferences (67). Each recommendation will generate three different options, and users can further manually adjust the meal combinations during the browsing process, and view the modified ratings and nutritional balance in real time. This mechanism not only ensures the adaptability of the recommendation results to most common dietary patterns, but also provides flexibility for individual differences, thereby enhancing the robustness and universality of the recommendation system.

### 4.2 System evaluation

#### 4.2.1 Group recommendation result

The group recommendation experiment compared the nutritional quality of dietary plans between real-life scenarios and system-generated recommendations. The real-life scenario data was derived from 30 days of lunch data from a primary school cafeteria, designed and configured by a part-time nutritionist, and adhered to established nutritional health standards. The recommendation data was generated from 30 days of lunch plans automatically recommended by the system for users aged 9–11. The following analysis compared the two approaches across three dimensions: overall dietary quality, nutritional balance, and food diversity.

**Figure 5:**
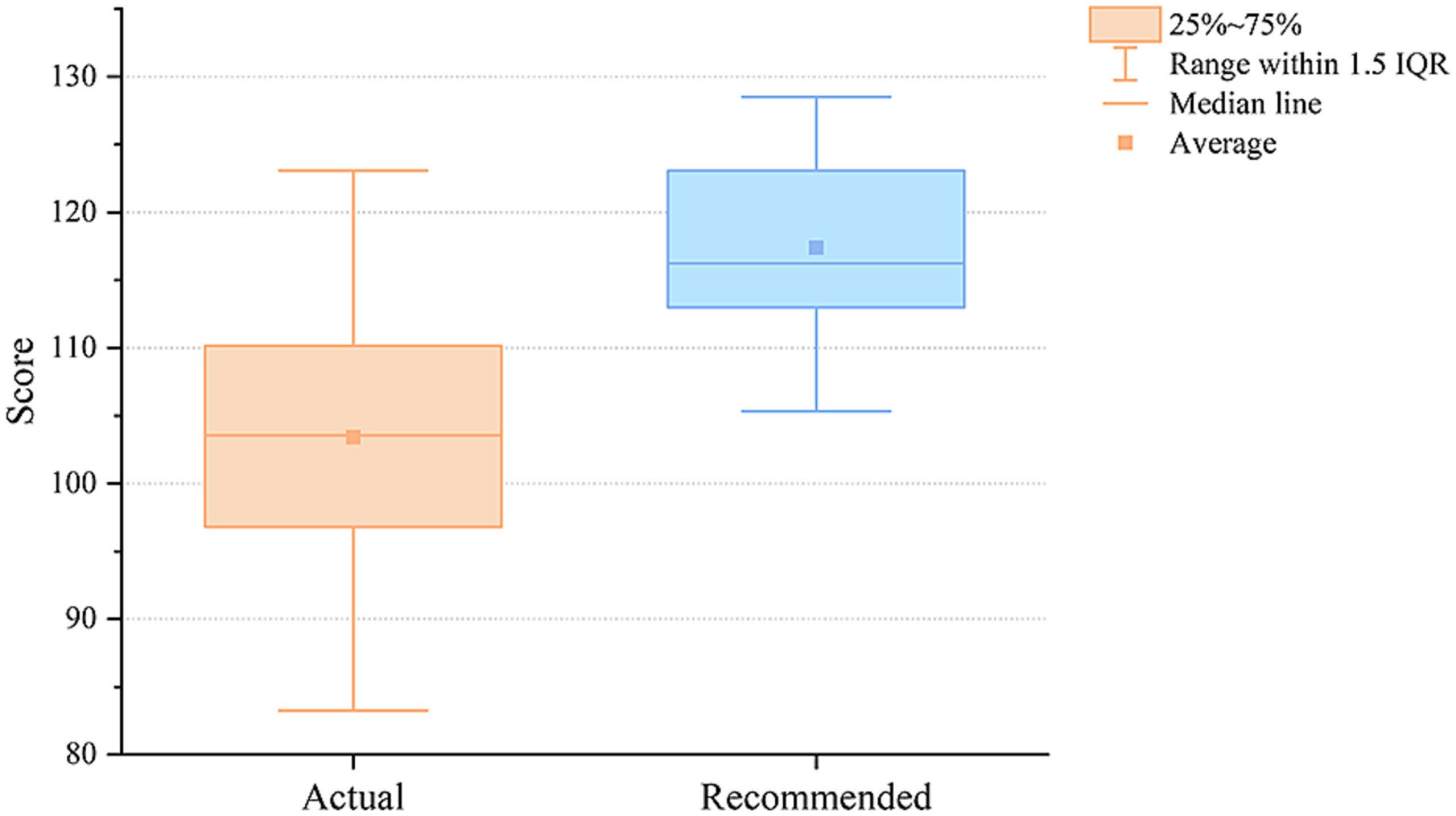
The 30-day index distribution for actual and recommended meal plans in the group recommendation experiment.

Comprehensive Quality of Diet: Figure 5 illustrates the trend of Chinese Children Dietary Index (CCDI) scores (68) for both actual and recommended meal plans over a 30-day period in the primary school cafeteria. The horizontal axis represents time in days, and the vertical axis denotes the CCDI score. The plotted points correspond to the daily lunch scores, and the dashed line indicates the average score over the period. The upper blue line represents the recommended meal plans, which have an average score of 117.39 ± 6.61 points and a quartile range M (P25, P75) of 116.23 (112.55, 122.98). The lower orange line corresponds to the actual meal plans, with an average score of 103.41 ± 9.44 points and a quartile range M (P25, P75) of 103.56 (96.27, 110.04). It is evident that the quality of the recommended meal plans is superior to that of the actual meal plans, with greater stability in the scores.

**Figure 6:**
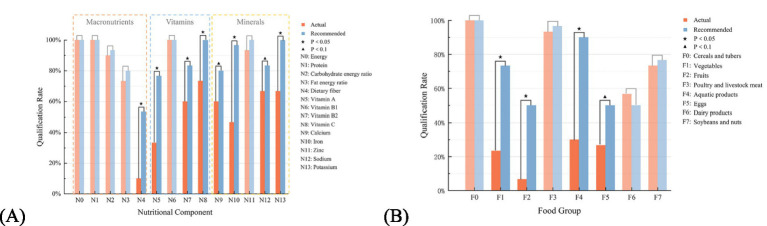
The qualification rates for actual and recommended meal plans in the group recommendation experiment: **(A)** Nutritional components; **(B)** food groups.

Nutritional Balance: Figure 6A illustrates the qualification rates of 14 nutritional components in both actual and recommended meal plans over a 30-day period in the primary school cafeteria. Certain nutrients, including energy, fat energy ratio, carbohydrate energy ratio, and sodium, should be consumed in moderate amounts. For a meal plan to be considered as meeting the targets, the corresponding levels of these nutrients must fall within the reference ranges outlined in the dietary guidelines. For other nutrients, qualification is defined as meeting at least the lower limit of the reference range. The figure indicates that the qualification rates for energy, protein, carbohydrate energy ratio, fat energy ratio, vitamin B1, and zinc are comparable between the actual and recommended meal plans, as represented by the lighter shaded areas. The recommended meal plans exhibit superior performance in other nutrients, including dietary fiber, vitamin A, vitamin B2, vitamin C, calcium, iron, sodium, and potassium, showing significant differences when compared to the actual meal plans. However, the qualification rates for dietary fiber and calcium in the recommended meal plans remain insufficient, potentially influenced by the meal library and dietary habits. Future considerations should focus on increasing the availability of related meals and enhancing awareness. Additionally, the absence of oil and salt data in the cafeteria’s collected meal data may introduce bias in the sodium assessment, although its impact on the comparative analysis of the two meal plans is relatively minimal.

In calculating the number of qualified nutrients for each meal plan, a total of 14 nutrients were taken into account. The actual meal plans contain an average of 7.47 ± 1.85 qualified nutrients, whereas the recommended meal plans show an average of 9.13 ± 0.13. Based on the above data, it can be concluded that the nutritional balance of the recommended meal plans not only meets but exceeds that of the actual meal plans in certain nutrients, thereby improving the provision of nutrients that are deficient in the actual meal plans.

Food Diversity: Figure 6B illustrates the qualification rates of various food groups in both the actual and recommended meal plans over a 30-day period in the primary school cafeteria. A food group is deemed qualified if its intake exceeds the dietary target reference value. The figure indicates that the qualification rates for cereals and tubers, poultry and livestock meat, dairy products, and soybeans and nuts are comparable between the actual and recommended meal plans, as represented by the lighter shaded areas. The recommended meal plans outperform the actual meal plans in the categories of vegetables, fruits, aquatic products, and eggs, with particularly significant differences in the provision of vegetables and aquatic products, but the qualification rates for vegetables, eggs, fruits, and dairy products in the recommended meal plans remain suboptimal. This may be partly attributed to considering only lunch meals and the limitations of the meal library. Additionally, in the recommendation algorithm, fruits and dairy products are not strictly designed to meet the guideline of approximately 200 grams per day, but are adjusted based on actual dining habits and economic constraints, resulting in a deviation from the reference intake. Analysis of the actual meal plans reveals a higher frequency of dairy product provision compared to fruits, which leads to the observed differences in qualification rates for fruits and dairy products between the actual and recommended meal plans. Data on oil and salt could not be included in the analysis because they were not available in the cafeteria meal records collected.

In calculating the number of qualified food groups for each meal plan, with a total of 8 food groups considered (excluding oil and salt due to lack of data), the actual meal plans contain an average of 3.87 ± 0.94 qualified food groups, and the recommended meal plans contain an average of 4.69 ± 0.88. A Wilcoxon signed-rank test was then performed to compare the two groups. The results are presented in Table 1. Based on this data, it can be concluded that the food diversity of the recommended meal plans not only meets but surpasses that of the actual meal plans in some food groups, improving the supply of those lacking in the actual plans. Considering the dietary guidelines for diversity, including a mix of coarse and refined grains and dark-colored vegetables, the average amount of coarse grains in the cereals and tubers category is 3.66 grams in the actual meal plans, compared to 52.98 grams in the recommended meal plans. In the vegetables category, the average amount of dark-colored vegetables is 79.89 grams in the actual meal plans and 120.17 grams in the recommended meal plans. The recommended system clearly outperforms in balancing coarse and refined grains in staple foods and in enhancing the nutritional quality of vegetables. This is further reflected in the improved intake of dietary fiber, vitamin A, and other micronutrients as shown in the nutritional balance assessment.

**Table 1:** Statistical test results for various indicators between actual and recommended meal plans in the group recommendation experiment.

Indicators	Actual meals	Recommended meals	*p*-value
CCDI	103.41 ± 9.44	117.39 ± 6.61	<0.001
Average number of qualified nutrient (pcs/14)	7.47 ± 1.85	9.13 ± 0.13	<0.001
Average number of qualified food group (pcs/8)	3.87 ± 0.94	4.69 ± 0.88	<0.001

#### 4.2.2 Individual recommendation result

The individual recommendation experiment compared the performance of dietary plans in terms of personalized nutritional health between real-life scenarios and system-generated recommendations. The real-life scenario data was derived from the physical examination results and 30 days of full-day dining records of 30 users from a middle school. The recommendation data consisted of 30-day full-day meal plans, individually generated by the system based on the personalized dietary goals of each user. Table 2 shows some of the physical examination data for these 30 students. A comparative analysis of the two datasets was conducted across three dimensions: comprehensive diet quality, nutritional balance, and food diversity.

**Table 2:** Statistical tables of partial physical examination data of students in individual experiments.

Entry	Feature	Number of cases	Ratio
Gender	Male	18	60.00%
	Female	12	40.00%
Nutritional Status	Normal	19	63.33%
	Malnutrition	2	6.67%
	Overweight	6	20.00%
	Obesity	3	10.00%
Blood Pressure	Normal	27	90.00%
	Elevated	3	10.00%
Vision	Normal	2	6.67%
	Mild impairment	1	3.33%
	Moderate impairment	6	20.00%
	Severe impairment	21	70.00%
Anemia	Normal	25	83.33%
	Mild anemia	5	16.67%
Other Medical History	Allergies	2	6.67%
	Asthma	1	3.33%

**Figure 7:**
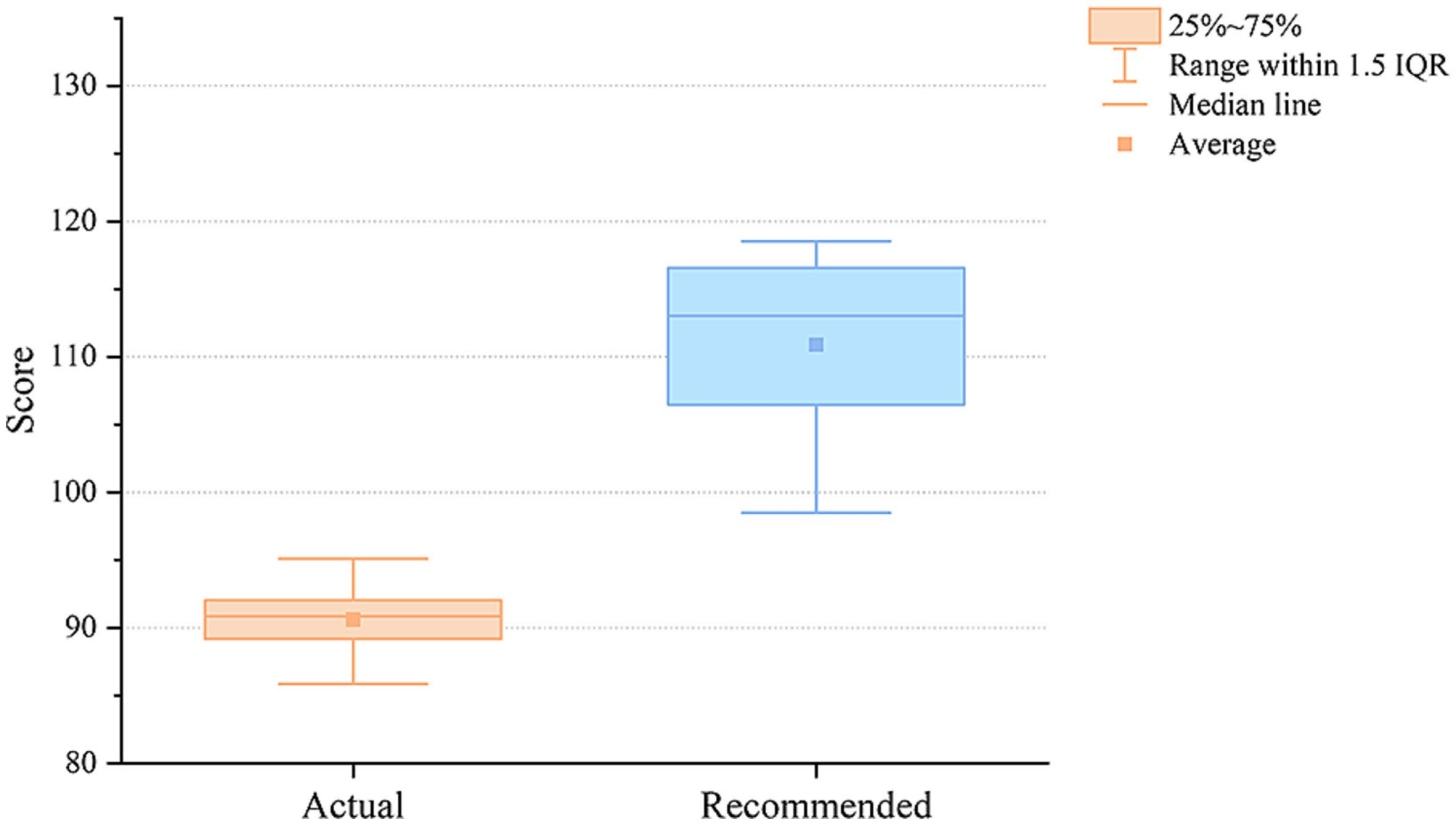
The average index distribution over 30 days for the self-selected and recommended meal plans of 30 users in the individual recommendation experiment.

Comprehensive Quality of the Diet: The calculation of the CCDI incorporates specific indicators tailored to the user’s dietary goals, reflecting the comprehensive dietary quality for different individuals. The overall average provides an indication of the comprehensive dietary quality of both the actual and recommended meal plans in relation to personalized nutritional health. The average CCDI score for the recommended meal plans is 110.91 points with a quartile range M (P25, P75) of 113.04 (106.41, 116.23), and the self-selected meal plans average 90.61 points with a quartile range M (P25, P75) of 90.86 (89.11, 91.82). Figure 7 illustrates that the quality of the recommended meal plans far exceeds that of the self-selected meal plans.

**Figure 8:**
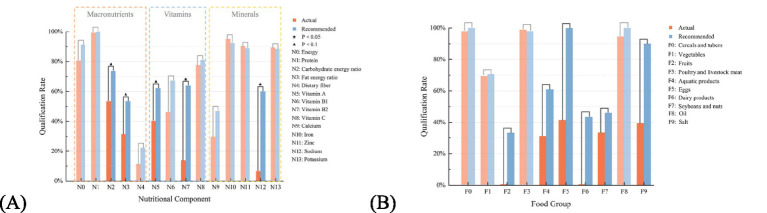
The qualification rates for actual and recommended meal plans in the individual recommendation experiment: **(A)** Nutritional components; **(B)** food groups.

Nutritional Balance: Figure 8A depicts the qualification rate of various nutrients in the 30-day meal plans of 30 users for both self-selected and system-recommended meals. The criteria for determining nutrient qualification may vary among users depending on their personalized dietary goals. Calculating the number of qualified nutrients per meal plan, considering a total of 14 nutrients, shows that the average number of qualified nutrients in the self-selected meal plans is 7.43 ± 1.80, whereas in the recommended meal plans, it is 8.89 ± 1.96. These results indicate that, in terms of nutritional balance, the recommended meal plans outperform the self-selected ones, particularly in improving the intake of nutrients that were deficient in the self-selected plans.

Food Diversity: Figure 8B illustrates the qualification rate of various food groups in the 30-day meal plans of 30 users for both self-selected and recommended meals. The criteria for determining the qualification of a food group may vary among users depending on their personalized dietary goals. The number of qualified food groups per meal plan is calculated based on a total of 10 food groups. The average number of qualified food groups in the self-selected meal plans is 4.98 ± 1.02, whereas in the recommended meal plans, it is 6.81 ± 0.86. These findings indicate that in terms of food diversity, the recommended meal plans outperform the self-selected ones, particularly in improving the intake of food groups that were deficient in the self-selected plans.

The mean and standard deviation for each of the 30 users were calculated for CCDI, the number of qualified nutrients, and the number of qualified food groups. A Wilcoxon signed-rank test was then performed to compare the two groups. The results are presented in Table 3. The statistical test results further support the conclusions drawn from the preceding analysis.

**Table 3:** Statistical test results for key indicators between self-selected and recommended meal plans in the individual recommendation experiment.

Indicators	Self-selected meals	Recommended meals	*p*-value
CCDI	90.61 ± 10.77	110.91 ± 9.7	<0.001
Average number of qualified nutrients (pcs/14)	7.43 ± 1.80	8.89 ± 1.96	<0.001
Average number of qualified food group (pcs/10)	4.98 ± 1.02	6.81 ± 0.86	<0.001

## 5 Discussion

### 5.1 Principal findings

This study developed an integrated framework for dietary recommendations tailored to children and adolescents. By combining ontology construction, a multi-criteria decision-making algorithm, and a multi-scenario recommendation system, we addressed the unique population characteristics, nutritional requirements, and dining contexts of this demographic. Experimental validation confirmed that the proposed system can effectively generate meal plans with superior nutritional quality, balance, and diversity compared with actual diets.

Firstly, we constructed a dietary recommendation ontology by integrating physical examination data, dietary guidelines, field surveys and expert advice. Using a structured methodology, we defined and instantiated relevant concepts and relationships, thereby establishing an ontology that captures both population characteristics and dining scenarios. This ontology provides a robust knowledge base for subsequent algorithm and system development.

Secondly, we designed a dietary recommendation algorithm grounded in multi-criteria decision-making. By integrating multi-attribute and multi-objective approaches, the algorithm addresses the complex nutritional demands of children and adolescents. It comprises three stages: meal combination guided by specific rules, portion calculation using a genetic algorithm, and program selection through a distance-based method. This enables the generation of personalized meal plans that balance nutrient adequacy and food diversity.

On the basis of ontology and algorithm, we developed a multi-scenario dietary recommendation system for school meal services and family dining. The system architecture integrates the ontology knowledge base and algorithm on the server side, with user-oriented terminals designed for different dining contexts. Together, these components enable dietary goal reasoning, meal set management, personalized plan generation, and comprehensive dining data management.

System performance was evaluated through both group and individual experiments. Results demonstrated that the system-generated meal plans outperformed actual diets in nutritional quality, dietary balance, and food diversity. These findings validate the system’s accuracy, feasibility, and personalization capacity, while also identifying areas where the knowledge base and algorithms could be further refined.

### 5.2 Interpretation of the results

In both the school and home meal scenarios, CADRS-generated plans not only met the dietary goals but consistently outperformed real-world counterparts on overall dietary quality, nutritional balance and food diversity. These are not three separable accomplishments but three facets of the same underlying mechanism: an ontology that operationalizes dietary guidelines as structured rules and a multi-criteria decision pipeline that turns those rules into joint optimization targets. Each of the three observed outcomes can be traced to a specific component of this pipeline.

The CCDI scores most directly capture overall dietary quality. The system was able to evaluate every candidate meal against more than ten interdependent indicators simultaneously. This level of co-optimization is cognitively prohibitive in manual planning. The result was a higher and notably more stable CCDI distribution: 117.39 ± 6.61 against 103.41 ± 9.44 in the school setting, and 110.91 ± 9.70 against 90.61 ± 10.77 at the individual level (both *p* < 0.001). The narrower variance is itself informative, it indicates that the system raised the floor of dietary quality across the 30-day period, not merely the average.

The observed gains in nutritional balance came from the MOEA/D-based portion-optimization step, which works in the joint space of 14 nutrient objectives under per-dish weight constraints. Because trade-offs between nutrients are resolved within a single multi-objective search rather than addressed sequentially, the recommended plans achieved simultaneous improvements that are difficult to obtain heuristically: the qualification rates of dietary fiber, vitamin A, vitamin B2, vitamin C, calcium, etc. improved simultaneously, lifting the average count of qualifying nutrients from 7.47 ± 1.85 to 9.13 ± 0.13 in the school experiment and from 7.43 ± 1.80 to 8.89 ± 1.96 at the individual level.

For food diversity, the distance-based selection step on the Pareto-optimal frontier proved decisive. Coverage across the ten dietary-pagoda groups was treated as a co-equal objective rather than a soft constraint, so the final solutions were chosen explicitly to minimize the deviation from ideal diversity. This is reflected not only in the higher count of qualifying food groups (from 3.87 to 4.69 in the group experiment, *p* < 0.001; from 4.98 to 6.81 at the individual level, *p* < 0.001) but also in the composition of specific categories: average daily intake of coarse grains rose from 3.66 g to 52.98 g and that of dark-colored vegetables from 79.89 g to 120.17 g. These are guideline-recommended sub-targets that are routinely under-supplied in real-world Chinese pediatric diets.

A few categories nevertheless remained below guideline targets, notably dietary fiber and calcium among the nutrients and fruits, dairy products, soybeans and nuts among the food groups. Two factors contribute to this: the meal database under-represents dishes rich in fiber, soy and nuts, and the algorithm permits day-to-day alternation of fruits and dairy rather than imposing daily targets strictly, in order to remain compatible with prevailing eating habits and household economics. Both can be addressed in future iterations by enriching the meal library and by introducing softer, weekly-rolling diversity constraints.

### 5.3 Comparison with realistic scenario

To further illustrate the contribution of this study, we selected two real-world dietary surveys conducted in Fujian Province and Yunnan Province, representing the dietary and nutritional status of Eastern and Western China, for comparison with the proposed dietary recommendation system. Since these surveys employed a 3-day 24-h dietary recall method targeting children and adolescents within the regions, direct comparisons regarding nutritional adequacy and food diversity compliance between the surveyed diets and the 30-day lunch menus generated by our recommendation system were not feasible. Therefore, we compared key outcome metrics from the surveys with the CCDI scores derived from our group recommendation experiments to evaluate differences in overall dietary quality between the system-generated menus and actual dietary patterns in these regions. The detailed description of the two studies can be found in Supplementary File S4.

The first study investigates the dietary quality of primary and middle school students in Yunnan Province (69). It utilized a stratified cluster random sampling approach, selecting 1,078 students from six prefecture-level cities in Yunnan Province between August and November 2022 as participants. Based on a 3-day 24-h dietary recall, the dietary quality of the participants was assessed using the CCDI. The results revealed that the total CCDI score M (P25, P75) for children and adolescents in urban areas of Yunnan Province was 65.30 (54.84, 73.62), while the score for rural areas was 62.17 (54.31, 70.70). These scores are significantly lower than the quartile range of 116.23 (112.55, 122.98) observed in the experimental group recommended in this study.

The second study examines the dietary quality of children and adolescents aged 7–17 in Fujian Province (70). It utilized a multi-stage stratified random sampling approach to select school-age children from nine prefecture-level cities within the province. Dietary quality was assessed using the CCDI based on dietary surveys conducted through the 3-day 24-h recall method. Disparities across demographic characteristics were also analyzed. The final results indicated that the CCDI score M (P25, P75) for adolescents in urban areas of the province was 65.93 (28.04, 97.59). In rural areas, the CCDI score was 59.18 (26.12, 96.70). Both urban and rural scores were lower than the CCDI scores observed in the experimental group recommended in this study.

Several important caveats should be noted when interpreting this cross-study comparison. First, the comparison is indirect: our system-generated CCDI scores were derived from 30-day computationally optimized lunch menus, whereas the Fujian and Yunnan scores were based on 3-day 24-h dietary recalls of actual intake, which differ in assessment methodology, time span, and the number of meals covered. Second, our system validation was conducted using meal data from two schools in Zhejiang Province. These methodological and geographic limitations preclude definitive conclusions about regional applicability.

With these caveats in mind, the published survey data suggest that the overall dietary quality of children and adolescents in both the eastern (Fujian) and western (Yunnan) regions is suboptimal, with CCDI scores considerably lower than those achieved by our system-generated meal plans. Dietary quality in rural areas is generally lower than in urban areas. The meal plans generated by the proposed recommendation system in this study consistently achieved substantially higher comprehensive dietary quality scores compared to the two real-world dining scenarios, suggesting its potential practical value. Specifically, children and adolescents in both regions show pronounced inadequacies in the intake of fruits and dairy products, coupled with the overconsumption of poultry products. Additionally, children and adolescents in Yunnan Province show a pronounced deficit in seafood intake, which is likely influenced by local dietary habits. In contrast, the system-generated meal plans demonstrated better performance in these food categories, suggesting the potential to mitigate the extreme overconsumption or deficiency of any specific food type or nutrient. These observations provide preliminary evidence that the recommendation system may help improve dietary quality, address imbalances in real-world dietary scenarios and facilitates the reduction of regional disparities in dietary quality, particularly in economically underdeveloped areas or among populations with relatively monotonous dietary habits.

### 5.4 Strengths and implications

Our study presents several distinct strengths. First, this study constructs a comprehensive ontology of dietary recommendations for children and adolescents, which not only covers the analysis of physical examination data and the compilation of guideline literature, but also ensures the scientific and practicality of the recommended content through fieldwork and expert advice. It supports the precise definition of 177 concepts with multidimensional attributes, facilitating a comprehensive and nuanced understanding of dietary requirements.

Secondly, CADRA differs from prior dietary-optimization work in its overall architecture rather than in parameter tuning. Most existing linear and integer programming formulations operate at the food-group or ingredient level and cannot natively express dish-level combinatorial structure, and conventional content- or collaborative-filtering recommenders provide no built-in mechanism for population- or condition-specific reasoning unless they are substantially augmented with external nutritional knowledge bases or rule modules. CADRA replaces these with an ontology-driven, dish-level rule engine that encodes Chinese culinary logic into executable composition, an MOEA/D portion optimizer that resolves 14 nutrient objectives jointly, and a distance-based selection step in which food-group diversity acts as a co-equal criterion rather than a constraint. Age-stratified Dietary Reference Intakes and condition-specific priorities are inherited directly from the ontology and propagate into the objective function, instead of being applied as post-hoc filters. The CCDI averages of 117.39 and 110.91 in the group and individual experiments both well above human-designed counterparts, and the food diversity and nutritional balance were significantly improved.

In addition, the multi-scenario meal recommendation system for children and adolescents developed realizes comprehensive coverage of home and school meal scenarios through the design of the server and two user terminals. The system mainly targets two types of users: (1) nutritionists, teachers and cafeteria managers in the school meal scenario, and (2) parents and children in the family meal scenario. The former is concerned about how to develop nutritionally balanced collective meal plans under limited budget and manpower, while the latter expects to get convenient and personalized healthy dietary advice. In typical application scenarios, the system can serve both centralized school meal environments, supporting standardized nutritional lunch designs, and daily family meal management, offering personalized recommendations and adjustments. This dual-scenario coverage allows the system to meet multi-level needs from groups to individuals, enhancing its practicality and scalability. In terms of adoption factors, ease of use and cost-effectiveness are key drivers. To lower the usage threshold, the family side adopts a WeChat Mini Program, which allows users to obtain dietary advice at any time without installing additional applications, and this “ready-to-use” feature fits the usage habits of parents and teenagers; while the school side provides efficient data management and recommendation interfaces through the web terminal, which facilitates the seamless integration with the existing meal supply process. On the cost side, the system emphasizes compatibility with existing infrastructure without adding additional hardware or high maintenance fees, enabling schools to implement the system without significant financial or manpower burdens, while the family side provides free basic recommendation services, further reducing the resistance to promotion and adoption.

### 5.5 Limitations and future work

This study has certain limitations, and future work can be expanded and deepened in the following areas to enhance its quality:

First, the dietary recommendation ontology and database can be further expanded to enhance the system’s representativeness. Although the current pilot study involving 30 students and 1 school cafeteria over 30 days provides essential preliminary evidence in a real-world school setting, we recognize that a broader user survey across multiple regions is required. Such expansion will help refine user profiling for Chinese children and adolescents and ensure the statistical generalizability of the findings on a larger scale. And the meal database can also be expanded to include categories such as beverages and snacks, while also considering additives and processing methods, evaluating their impact on dietary nutrition, so as to achieve more comprehensive daily diet optimization and more personalized and responsive meal recommendations.

Second, the dietary recommendation algorithm could be further optimized. Evaluation experiments of the dietary recommendation system revealed a gap between the nutrient or food group quantities in the recommended meal plans and the guideline references. Further analysis is needed to identify the root causes of this issue, and collaborate closely with nutrition experts to explore optimization and improvement strategies. Attempts can also be made to introduce more advanced optimization methods, such as deep learning or reinforcement learning, to improve the computational efficiency and generalization ability in large-scale meal databases.

Third, the scientific rigor and generalizability of the recommendation system could be further strengthened through standardized benchmarking and cross-cultural localization. Currently, the absence of established baselines that accommodate the complex multi-dish structure of Chinese meals makes comparative evaluation challenging. Future work should prioritize the development of evaluation benchmarks to quantify the system’s performance relative to other international health recommender systems, while also adapting the ontology and data sources for non-Chinese dietary culture contexts. In addition, nutritional science itself is constantly evolving, and the definition of a healthy diet is constantly being adjusted as new research findings emerge. To enhance the scientific validity and foresight of the system in long-term use, the key knowledge modules in the system can be iteratively updated based on the latest dietary guidelines, epidemiological findings or expert consensus. Nutritional goals and constraints in the recommendation algorithm can also be flexibly updated based on the adjustment of the knowledge base, to ensure that the recommendation results remain aligned with the latest scientific knowledge.

Fourth, scaling the system from a single-school pilot to multi-school, multi-region deployment presents several practical challenges that warrant further investigation. Data heterogeneity is a primary concern: meal libraries, food composition data, and student health records vary substantially across schools and regions in format, granularity, and completeness, necessitating robust data harmonization and quality assurance pipelines. Infrastructure variability also poses challenges, as schools in economically underdeveloped areas may lack reliable internet connectivity or the technical capacity to maintain digital nutrition management systems, which calls for lightweight deployment solutions and offline-capable functionalities. Furthermore, effective large-scale deployment requires multi-stakeholder coordination among schools, local education bureaus, health authorities, and parents, each with distinct priorities and constraints. Future work should explore governance frameworks and collaborative models—such as public-private partnerships or government-led pilot programs—that can facilitate sustainable adoption. Addressing these challenges through targeted pilot studies in diverse settings would generate the empirical evidence needed to support policy recommendations for nationwide implementation.

## 6 Conclusion

This study successfully constructed a dietary recommendation ontology for children and adolescents and developed an algorithm and system based on multi-criteria decision-making, enabling the generation of personalized dietary plans. Experimental evaluations using real-world data from schools in Zhejiang Province demonstrated the system’s high potential feasibility and accuracy in both family and school settings, indicating its effectiveness in improving the nutritional health of children and adolescents and promoting healthy growth, demonstrating significant social importance and application value. Future research could expand the ontology’s content and optimize the algorithm to align more closely with dietary guidelines, and pursue multi-school, multi-region deployment through standardized deployment protocols, ontology localization, and integration with government nutrition surveillance frameworks, thereby translating the validated prototype into a scalable policy tool for improving child and adolescent dietary health nationwide.

## Data Availability

The raw data supporting the conclusions of this article will be made available by the authors, without undue reservation.
