# Supplementary material for: Ontology-based dietary recommendation system for Chinese children and adolescents: development and a pilot validation study
Source: Front Public Health. 2026 May 22;14:1780898. doi: 10.3389/fpubh.2026.1780898 (PMC13236952; doi:10.3389/fpubh.2026.1780898)
Supplement: Supplementary file 1 [file Table_1.DOCX]

Key Concepts of Dietary Health

**Supplementary Table 1:** Definitions of Entity Concepts for the Dietary Health Module

| **Concept** | **Definition** | **Example** |
| --- | --- | --- |
| Food | A wide range of edible ingredients and pre-processed foods with accurate nutrient content data | Pork, Eggplant |
| Ingredients | Basic foods for daily use | Pork belly |
| Dishes | A dish that consists of a variety of foods that have been processed and cooked. | Tomato scrambled eggs |
| Set Meal | A combination of a number of dishes in a certain ratio, which can represent a certain meal of the day. | Fish-flavored shredded pork set meal includes white rice,  Fish-flavored shredded pork, stir-fried celery |
| One-day Set Meal | Includes three basic meals: breakfast, lunch, and dinner | One day's three meals include breakfast soy milk  and fried dough sticks set, lunch fish-flavored shredded pork set, dinner braised prawns set |
| Nutritional Components | Nutrients and beneficial components in food | Carbohydrates, proteins, etc. |
| Food Classification | According to the principle of two-level classification of food groups and subgroups in the Chinese Food Composition Table, there are 21 secondary groups under it, and 93 tertiary groups under the secondary groups. | Wheat, rice, corn, barley, etc. |
| Dietary Pagoda Food Group | According to the principles of food categorization proposed in the Dietary Guidelines for Chinese Residents, there are 10 secondary categories under them. | Cereals, vegetables, fruits, etc. |

**Supplementary Table 2:** Definition of Attribute Concepts for the Dietary Health Module

| **Concept** | **Definition** | **Range** |
| --- | --- | --- |
| Edible Part | Edible proportion in food | Numeric, minimum value is 0, maximum value is 1 |
| Food Color | Appearance and color of vegetables and their products, as well as fungi and algae-based foods | Light color, dark green, orange, red, purple, black |
| Grain Type | Processing characteristics of grains and their products, tuber starch and its products | Coarse grains, fine grains |
| Dish Type | Definitions based on the weights of animal and vegetable proportions of the constituent foods | Coarse grain staple, fine grain staple, mixed staple, main meat, semi-meat, vegetarian, soup, milk, fruit, others |
| Meal Time | Representative periods of the day when meals are consumed | Breakfast, lunch, dinner |
| Composition Type | Sum of the meal types of all meals combined into a set meal | In the form of "a meat b vegetarian c staple d others", where a, b, c, d are all integer values, minimum value is 0 |
