# Supplementary material for: Ontology-based dietary recommendation system for Chinese children and adolescents: development and a pilot validation study
Source: Front Public Health. 2026 May 22;14:1780898. doi: 10.3389/fpubh.2026.1780898 (PMC13236952; doi:10.3389/fpubh.2026.1780898)
Supplement: Supplementary file 2 [file Table_2.DOCX]

Rules for Dish Combination

# Rules for Single Set Meal Combination

1. Staple Food Pairing:

The selection of staple foods should align with the attributes of the meal type. Random choices can be made from the following pairing options: coarse-grain staple + refined-grain staple, refined-grain staple, or mixed-grain staple.

1. Non-Repetition of Meat Dishes:

A single meal may include one main meat dish and one semi-meat dish. To ensure diversity, the main ingredient categories of the main and semi-meat dishes must differ. This pairing should correspond to the meal type attributes and the primary ingredient’s food category.

1. Non-Repetition of Vegetarian Dishes:

A single meal may include up to two vegetarian dishes. The primary ingredients of these two dishes must be from distinct food categories to avoid redundancy. The selection should align with the meal type attributes and the primary ingredient attributes.

# Rules for One-Day Set Meal Combination

Building upon the requirements for single-meal combinations, the following additional rules govern daily meal combinations:

1. Non-Repetition of Primary Ingredients:

The primary ingredients across all meals in a day must be distinct. This selection must align with the meal type and primary ingredient attributes.

1. Dark Green Vegetable Requirement:

Dark green vegetables, which are rich in various vitamins and highly nutritious, must account for at least 50% of the total vegetable weight provided in a day. This proportion corresponds to the food color attribute.

1. Coarse Grain Requirement:

Coarse grains, which contain essential nutrients not found in refined grains, must be included in a specified quantity daily. This requirement aligns with the grain type attribute.

1. Food Variety Requirement:d

Food diversity is a fundamental dietary principle. Each day must include at least 12 distinct food items. This count corresponds to the food category attribute and excludes seasonings such as salt, sugar, and oil from the total food variety calculation.

# Rules for One-Week Set Meal Combination

Building upon the principles for single-meal and daily meal combinations, the following additional rules apply to weekly meal planning:

1. Special Food Category Requirement:

Special food categories provide unique nutritional benefits and should be included regularly in appropriate amounts. Over the course of a week, meals must include seafood, Soy products, Edible fungi, or algae at least 3 times respectively, which correspond to the food category attribute.

1. Food Variety Requirement:

A minimum of 25 distinct food items must be provided over the week. The method of counting food types follows the same rules as outlined in the daily meal combination guidelines, excluding seasonings like salt, sugar, and oil from the total.
