# Supplementary material for: Ontology-based dietary recommendation system for Chinese children and adolescents: development and a pilot validation study
Source: Front Public Health. 2026 May 22;14:1780898. doi: 10.3389/fpubh.2026.1780898 (PMC13236952; doi:10.3389/fpubh.2026.1780898)
Supplement: Supplementary file 3 [file Table_3.DOCX]

Description of the Evaluation Indicators for the Experiment

# Comprehensive Dietary Quality

The Chinese Children Dietary Index (CCDI) is employed as the evaluation metric for comprehensive dietary quality. This index is based on the Dietary Guidelines for Chinese Residents (2016) and the Chinese Dietary Nutrient Reference Intakes (2013), tailored to the dietary characteristics of Chinese children and adolescents. The CCDI provides a systematic method to assess the dietary quality of this population group, comprising 16 indicators encompassing nutrient composition, food groups, and health-related behaviors. The total score is 160 points, with higher scores indicating better overall dietary quality among children and adolescents (1). Compared to other indices, the CCDI integrates both nutrient and food composition aspects, offering a more comprehensive evaluation of dietary quality. Notably, most indicators are standardized based on the reference intake of food per 1000 kcal of energy consumption, effectively balancing the translation between single-meal and daily dietary standards.

# Nutritional Balance

Nutritional balance refers to the appropriate proportion and quantity of various nutrients in a diet, ensuring neither excess nor deficiency, to meet the physiological and developmental needs of the body. It is evaluated using the following two indicators:

1. Qualification Rate of 14 Nutrients in 30-Day Meal Plans:

Based on the Dietary Knowledge Database, 14 key nutrients—such as energy, protein, carbohydrates, and fats—are considered. For each nutrient, the quantity in each meal plan is compared with the reference dietary intake target to assess qualification. The qualification rate for each nutrient is calculated as the proportion of meal plans meeting the standard over 30 days.

1. Average Number of Qualified Nutrients in 30-Day Meal Plans:

For each meal plan, the quantities of 14 nutrients are reviewed, and the number of compliant nutrients is counted. The average number of compliant nutrients is then calculated across the 30-day meal plans.

Higher values for these indicators indicate that the meal plans better achieve nutritional balance, ensuring the diet meets physiological and developmental needs effectively.

# Dietary Diversity

Dietary diversity refers to the variety of different foods or food groups consumed over a specific period. It is assessed using the following two indicators:

1. Qualification Rate of 14 Food Groups in 30-Day Meal Plans:

Based on the dietary recommendations from the Dietary Knowledge Database and the Dietary Pagoda, food groups are categorized into 10 groups: cereals and tubers, vegetables, fruits, livestock and poultry meat, aquatic products, eggs, dairy and dairy products, soy and nuts, oil, and salt. For each food group, the quantity in each meal plan is compared with the reference dietary intake target to determine whether it meets the standard. The qualification rate for each food group is calculated as the proportion of compliant meal plans over 30 days.

1. Average Number of Compliant Food Groups in 30-Day Meal Plans:

For each meal plan, the 10 food groups are reviewed, and the number of compliant food groups is counted. The average number of compliant food groups is then calculated across the 30-day meal plans.

Higher values for these indicators signify that the corresponding meal plans better fulfill dietary diversity requirements, emphasizing balanced and varied nutrition.

1. Qiao T, Duan R, Cheng G. Revision of the Chinese Children Dietary Index. Acta Nutr Sin. (2019) 41(2):105–9. doi:10.13325/j.cnki.acta.nutr.sin.2019.02.002
