# Supplementary material for: Ontology-based dietary recommendation system for Chinese children and adolescents: development and a pilot validation study
Source: Front Public Health. 2026 May 22;14:1780898. doi: 10.3389/fpubh.2026.1780898 (PMC13236952; doi:10.3389/fpubh.2026.1780898)
Supplement: Supplementary file 4 [file Table_4.DOCX]

Details of the Realistic Scenario Studies

# Evaluation of Dietary Quality of School-Age Children in Fujian Province (1)

This study conducted a comprehensive evaluation of the dietary quality of children and adolescents aged 7–17 in Fujian Province, utilizing the Chinese Children's Dietary Index (CCDI-2016) (2) as an assessment tool. The objective was to identify nutritional inadequacies and provide evidence-based recommendations for dietary interventions.

## Sampling and Participants

A multi-stage stratified random sampling method was employed to ensure representative data collection. The study selected participants from nine cities across Fujian Province, encompassing six monitoring points per city. At each monitoring point, two primary schools, two middle schools, and one high school were randomly chosen. Within each school, one class per grade (from grades 1 to 6, as well as junior high and senior high levels) was randomly sampled, and 28 students from each class (equally divided by gender) were included. This approach ensured diversity in demographic coverage, including age, gender, and urban-rural differences.

## Data Collection

The study utilized the 3-day, 24-hour dietary recall method to capture detailed dietary intake information. Participants were asked to recall all foods and beverages consumed over three consecutive days, which included two weekdays and one weekend day. For children under nine years of age, dietary information was provided by their guardians, while older children reported their intake directly. Trained investigators conducted face-to-face interviews to ensure accurate and consistent data collection.

## Dietary Assessment

The dietary data were analyzed using the CCDI-2016, a validated tool based on the "Chinese Dietary Guidelines (2016)" and the "Chinese Dietary Reference Intakes (2013)." The CCDI-2016 assesses dietary quality across 16 food and nutrient indicators, including cereals, vegetables, fruits, dairy products, beans, poultry and meat, aquatic products, eggs, vitamin A, dietary fiber, and sugar-sweetened beverages. Scores for each category ranged from 0 to 10, with higher scores indicating better compliance with dietary recommendations. The CCDI-2016 also incorporates metrics for energy balance and healthy eating behaviors, providing a comprehensive assessment of overall dietary quality.

## Data Analysis

Data were analyzed using SPSS software (version 21). Normality tests determined the appropriate statistical methods: normally distributed data were analyzed using t-tests and ANOVA, while non-normally distributed data were assessed using Wilcoxon rank-sum tests or Kruskal-Wallis tests. Subgroup analyses were performed to evaluate differences in dietary quality by gender, urban-rural status, and age groups (7–10, 11–13, and 14–17 years). All statistical tests were two-tailed, with a significance threshold of p < 0.05.

## Findings and Conclusion

The study revealed that while cereals, eggs, and aquatic products generally met recommended intake levels, significant deficiencies were observed in the consumption of vegetables, beans, fruits, and dairy products. Conversely, poultry and meat consumption exceeded recommended levels. The dietary quality was higher among urban children compared to their rural counterparts and higher in girls than boys. Age-related trends showed improvements in the intake of cereals and beans but a decline in vitamin A intake with age.

Overall, the average CCDI-2016 score was 66.7, indicating moderate dietary quality with substantial room for improvement. The study concluded that targeted interventions are necessary to address these nutritional gaps, emphasizing increased consumption of vegetables, fruits, and dairy products while reducing excessive poultry and meat intake. These findings provide critical epidemiological evidence to inform public health policies and nutritional education programs aimed at optimizing dietary habits among children and adolescents in Fujian Province.

The main tables of the study are as follows:

Table 1. Basic demographic characteristics

|  | n | Ratio(%) |
| --- | --- | --- |
| Sex |  |  |
| Boys | 263 | 48.3 |
| Girls | 282 | 51.7 |
| Area |  |  |
| City | 229 | 42 |
| Countryside | 316 | 58 |
| Age(years) |  |  |
| 7-10 | 255 | 46.8 |
| 11-13 | 154 | 28.3 |
| 14-17 | 136 | 25 |

Table 2. The consumption of various foods (Median percentile (25-75))

|  | Energy  (kcal/d) | Grains and tubers(g/d) | Vegetables  (g/d) | Fruits  (g/d) | Dairy  (g/d) | Soybeans  (g/d) | Poultry and meat(g/d) | Sea food | Eggs |
| --- | --- | --- | --- | --- | --- | --- | --- | --- | --- |
| Age(years) |  |  |  |  |  |  |  |  |  |
| Recommended intake | 1400-1600 | 175-250 | 300 | 150-200 | 300 | 105 | 40 | 40 | 25-40 |
| 7-10 | 1741  (1393-2075) | 231  (183-290) | 159  (92-242) | 11  (0-92) | 0  (0-141) | 5  (0-15) | 124  (74-204) | 26  (0-67) | 43  (21-71) |
| Recommended intake | 1800-2000 | 250-300 | 400-450 | 200-300 | 300 | 105 | 50 | 50 | 40-50 |
| 11-13 | 1585  (1265-1992) | 204  (160-261) | 155  (93-237) | 0  (0-67) | 0  (0-139) | 5  (0-14) | 130.5  (81-215) | 29  (0-67) | 35  (18-54) |
| Recommended intake | 2000-2400 | 300-400 | 450-500 | 300-350 | 300 | 105-175 | 50-75 | 50-75 | 50 |
| 14-17 | 1360  (1107-1726) | 182.5  (150.5-255) | 144.5  (97.5-235.5) | 0  (0-79) | 0  (0-128) | 6  (0-18.5) | 112.5  (63-175.5) | 30  (0-57) | 35  (18-51) |

Table 3. The actual range of indexes of foods recommended for children of different ages (Median percentile (25-75))

|  | Grains and tubers(g/d) | Vegetables  (g/d) | Fruits  (g/d) | Dairy  (g/d) | Soybeans  (g/d) | Poultry and meat(g/d) | Sea food | Eggs |
| --- | --- | --- | --- | --- | --- | --- | --- | --- |
| Age(years) |  |  |  |  |  |  |  |  |
| Recommended intake | 110-179 | 188-214 | 94-143 | 188-214 | 10-10.7 | 25-29 | 25-29 | 16-29 |
| 7-10 | 141.39  (108.21-168.64) | 88.84  (57.17-129.2) | 5.63  (0-52.98) | 0  (0-84.82) | 3.12  (0-9.33) | 74.73  (45.65-112.64) | 14.9  (0-38.46) | 24.79  (11.68-42.94) |
| Recommended intake | 125-167 | 200-250 | 100-167 | 150-167 | 7.5-8.3 | 25-28 | 20-28 | 20-28 |
| 11-13 | 133.64  (109.2-162.69) | 103.23  (66.54-154.36) | 0  (0-44.56) | 0  (0-81.15) | 3.18  (0-10.48) | 93.61  (56.83-121.6) | 18.02  (0-44.23) | 20.6  (10.75-37) |
| Recommended intake | 125-200 | 187.5-250 | 125-175 | 125-175 | 6-12.5 | 21-37.5 | 21-37.5 | 21-25 |
| 14-17 | 139.97  (107.64-172.57) | 103.61  (70.34-160.17) | 0  (0-54.65) | 0  (0-83.95) | 4.65  (0-12.34) | 81.79  (52.76-109) | 19.1  (0-40.15) | 24.34  (11.21-36.16) |

Table 4. Scores of various foods according to CCDI-16

|  | Median | Percentile (25-75) |
| --- | --- | --- |
| Energy balance | 8.16 | 6.20-9.69 |
| Grains and tubers | 8.70 | 6.89-9.88 |
| Vegetables | 5.11 | 3.39-7.52 |
| Fruits | 0 | 0-4.57 |
| Dairy | 0 | 0-5.41 |
| Soybeans | 4.12 | 0-10.00 |
| Poultry and meat | 0 | 0-2.17 |
| Sea food | 6.88 | 0-10.00 |
| Eggs | 7.09 | 2.01-10.00 |
| Sugar sweetened beverages | 10.00 | 10.00-10.00 |
| Dietary fiber | 5.99 | 4.52-7.87 |
| Vitamin A | 6.60 | 4.12-10.00 |

Table 5. Scores of various foods for Children with different demographic characteristics according to CCDI-16 (Median percentile (25-75))

|  | Grains and tubers | Vegetables | Fruits | Dairy | Soybeans | Poultry  and meat | Sea food | Eggs | Sugar sweetened beverages | Dietary  fiber | Vitamin A | Energy balance |
| --- | --- | --- | --- | --- | --- | --- | --- | --- | --- | --- | --- | --- |
| Sex |  |  |  |  |  |  |  |  |  |  |  |  |
| Boys | 8.66  (6.91-9.89) | 4.98  (3.04-7.6) | 0  (0-3.43) | 0  (0-6.26) | 3.06  (0-10) | 0  (0-0.52) | 6.88  (0-10) | 7.06  (2.56-10) | 10  (0-10) | 5.5  (4.01-7.48) | 6.6  (3.88-10) | 8.1  (5.82-9.63) |
| Girls | 8.71  (6.78-9.88) | 5.19  (3.57-7.52) | 1.52  (0-6.22) | 0  (0-4.99) | 5.47  (0-10) | 0  (0-3.44) | 6.86  (0-10) | 7.14  (0.08-10) | 10  (0-10) | 6.47  (4.88-8.12) | 6.59  (4.26-9.98) | 8.25  (6.61-9.75) |
| P | 0.95 | 0.717 | <0.001 | 0.308 | 0.003 | 0.021 | 0.883 | 0.821 | 0.151 | ＜0.001 | 0.894 | 0.154 |
| Area |  |  |  |  |  |  |  |  |  |  |  |  |
| Countryside | 8.76  (6.95-9.93) | 5.16  (2.86-8.44) | 0  (0-4.13) | 0  (0-4.94) | 4.07  (0-10) | 0  (0-2.61) | 4.54  (0-10) | 6.88  (2.18-10) | 10  (0-10) | 5.9  (4.28-7.69) | 5.66  (3.84-9.26) | 8.21  (6.01-9.7) |
| City | 8.62  (6.83-9.85) | 5.05  (3.59-6.91) | 0  (0-5.51) | 0.97  (0-5.65) | 4.3  (0-10) | 0  (0-1.87) | 8.5  (0.79-10) | 7.13  (1.62-10) | 10  (0-10) | 6.02  (4.62-8.12) | 7.22  (4.27-10) | 8.12  (6.32-9.68) |
| P | 0.467 | 0.666 | 0.43 | 0.188 | 0.914 | 0.426 | <0.001 | 0.817 | 0.234 | 0.232 | 0.007 | 0.634 |
| Age (years) |  |  |  |  |  |  |  |  |  |  |  |  |
| 7-10 | 7.39  (5.83-8.96) | 4.73  (3.04-6.87) | 0.6  (0-5.64) | 0  (0-4.51) | 3.12  (0-9.33) | 0  (0-3.44) | 5.96  (0-10) | 6.57  (0.31-10) | 10  (0-10) | 6.02  (4.25-7.69) | 8.28  (5.1-10) | 8.63  (6.57-9.97) |
| 11-13 | 9.3  (7.79-10) | 5.16  (3.33-7.72) | 0  (0-4.46) | 0  (0-5.41) | 4.24  (0-10) | 0  (0-0) | 7.21  (0-10) | 6.85  (3.16-9.82) | 10  (0-10) | 5.79  (4.4-7.47) | 5.53  (3.45-8.82) | 8.49  (6.98-9.74) |
| 14-17 | 10  (8.21-10) | 5.53  (3.75-8.54) | 0  (0-4.37) | 0  (0-6.72) | 7.76  (0-10) | 0  (0-1.81) | 9.1  (0-10) | 8.03  (2.49-10) | 10  (0-10) | 6.29  (4.95-8.54) | 5.5  (3.46-7.71) | 6.61  (5.22-8.19) |
| P | ＜0.001 | 0.043 | 0.254 | 0.401 | 0.005 | 0.043 | 0.309 | 0.104 | 0.021 | 0.07 | ＜0.001 | ＜0.001 |

Table 6. The total score of children’s dietary indexes according to CCDI-16

|  | Total score | | T/F | P |
| --- | --- | --- | --- | --- |
|  | Mean | SD |  |  |
| Total | 66.7 | 12.28 |  |  |
| Sex |  |  | -2.634 | 0.009 |
| Boys | 65.28 | 12.69 |  |  |
| Girls | 68.03 | 11.76 |  |  |
| Area |  |  | -2.054 | 0.04 |
| Countryside | 65.44 | 11.85 |  |  |
| City | 67.62 | 12.53 |  |  |
| Age(years) |  |  | 2.201 | 0.112 |
| 7-10 | 65.85 | 12.44 |  |  |
| 11-13 | 66.47 | 12.13 |  |  |
| 14-17 | 68.56 | 12.04 |  |  |

# Diet quality of primary and secondary school students in Yunnan Province (3)

In order to understand the current situation of dietary nutrition of children and adolescents in Yunnan Province and their dietary nutritional problems, and to provide a theoretical basis for the targeted development of dietary nutritional interventions for them, the study investigated and comprehensively evaluated the dietary quality of students in elementary school and junior high schools in Yunnan Province, using the China Children's Dietary Index (CCDI-2016) as an assessment tool.

## Sampling and Participants

The study employed a stratified random sampling method to select 1,078 primary and secondary school students aged 7–17 years from six prefecture-level cities in Yunnan Province, China. Participants were drawn from both urban and rural areas, encompassing diverse ethnic groups, including Han, Wa, and Hani. The sampling process ensured representative coverage of demographic and geographic diversity. Consent was obtained from schools, students, and parents, with a questionnaire response rate of 93.66%.

## Data Collection

Data were collected from August to November 2022 through a 3-day 24-hour dietary recall method. Trained investigators conducted the surveys on two weekdays and one weekend day, documenting participants’ dietary intake. The data included detailed records of food types, quantities, and nutrient intake. Additional demographic and socioeconomic information was gathered to assess the influence of contextual factors on dietary behaviors.

## Dietary Assessment

Dietary quality was evaluated using the Chinese Children's Dietary Index (CCDI-2016), which assesses food intake, nutrient adequacy, and health-promoting behaviors. Scores for individual food groups and nutrients were calculated based on their alignment with recommended intake levels. The CCDI-2016 provides a comprehensive scoring system, with higher scores indicating better dietary quality. The dietary data were further categorized by age, gender, ethnicity, and parental education levels.

## Data Analysis

Data were analyzed using SPSS 26.0. Descriptive statistics were reported as medians (P25, P75) for non-normally distributed variables. Group comparisons were conducted using Mann-Whitney U tests for two groups and Kruskal-Wallis H tests for multiple groups. The influence of demographic factors on dietary scores was assessed, with significance set at P < 0.05.

## Findings and Conclusion

The study revealed significant dietary imbalances among children and adolescents in Yunnan Province. While cereal, egg, and sugary beverage consumption met recommended levels, vegetables, legumes, vitamin A, and dietary fiber intake were insufficient. Severe deficiencies were observed in fruit, dairy, and aquatic product consumption, while poultry intake exceeded recommendations. Subgroup analysis showed that children aged 11–13 years had the highest dietary quality, and those aged 14–17 years had the lowest. Girls outperformed boys, urban students scored higher than rural students, and dietary scores improved with higher parental education levels. Wa children exhibited the best dietary quality, while Hani children scored the lowest.

The findings highlight the need for targeted dietary interventions in Yunnan Province, emphasizing increased consumption of fruits, dairy, legumes, and aquatic products, along with reduced poultry intake. Recommendations include enhancing nutrition education, raising community awareness, and establishing robust dietary monitoring systems to improve the overall nutritional health of children and adolescents in the region.

The main tables of the study are as follows:

Table 7. Average Daily Intake of Various Food Groups for Children and Adolescents in Different Age Groups [M (P25, P75)]

| Age (Years) | Energy (kcal) | Cereals (g) | Vegetables (g) | Fruits (g) | Dairy (g) | Beans (g) | Poultry (g) | Aquatic Products (g) | Eggs (g) |
| --- | --- | --- | --- | --- | --- | --- | --- | --- | --- |
| 7–10 | 1468 (1124, 1901) | 233 (177, 283) | 150 (133, 250) | 0 (0, 26) | 0 (0, 33) | 3 (0, 16) | 100 (83, 170) | 33 (0, 67) | 40 (16, 65) |
| 11–13 | 1590 (1367, 1812) | 250 (207, 287) | 190 (133, 257) | 0 (0, 17) | 10 (0, 83) | 4 (0, 15) | 142 (97, 208) | 0 (0, 16) | 27 (20, 50) |
| 14–17 | 1428 (1210, 1723) | 253 (217, 293) | 130 (100, 200) | 0 (0, 24) | 0 (0, 40) | 4 (0, 17) | 133 (88, 183) | 0 (0, 27) | 26 (16, 44) |

Table 8. The Actual Index of Food Recommendation (IFR) for Children and Adolescents of Different Age Groups [(g·1000 kcal-1), M (P25, P75)]

| Age  (Years) | Cereals | Vegetables | Fruits | Dairy | Beans | Poultry | Aquatic Products | Eggs |
| --- | --- | --- | --- | --- | --- | --- | --- | --- |
| 7–10 | 157.96 (130.19, 203.99) | 118.90 (93.17, 142.00) | 0 (0, 21.22) | 0 (0, 31.44) | 2.65 (0, 8.47) | 83.33 (56.59, 110.55) | 15.41 (0, 47.26) | 24.06 (10.16, 47.31) |
| 11–13 | 156.79 (131.63, 188.60) | 115.25 (81.24, 163.23) | 0 (0, 11.90) | 5.59 (0, 46.81) | 2.47 (0, 8.07) | 94.24 (55.45, 120.13) | 0 (0, 14.48) | 17.54 (9.73, 31.41) |
| 14–17 | 169.44 (137.00, 221.04) | 92.19 (70.50, 123.19) | 0 (0, 16.70) | 0 (0, 25.84) | 2.50 (0, 7.65) | 96.41 (70.96, 116.96) | 0 (0, 17.71) | 19.74 (9.66, 34.64) |

Table 9. Dietary index scores of children and adolescents aged 7－17 in Yunnan Province for various food group [M (P25, P75)]

| Demographic Indicators | Options | Sample Size | Cereals | Vegetables | Fruits | Dairy | Beans | Poultry | Aquatic Products |
| --- | --- | --- | --- | --- | --- | --- | --- | --- | --- |
| Gender | Male | 497 | 9.89 (8.12, 10.00) | 5.39 (4.07, 7.54) | 0 (0, 1.15) | 0 (0, 1.49) | 3.32 (0, 10.00) | 0 (0, 1.37) | 0 (0, 8.79) |
|  | Female | 581 | 9.94 (8.35, 10.00) | 5.89 (4.19, 7.64) | 0 (0, 2.74) | 0 (0, 3.15) | 3.55 (0, 9.18) | 0 (0, 3.33) | 0 (0, 10.00) |
| Age(years) | 7–10 | 334 | 10.00 (8.05, 10.00) | 6.32 (4.96, 7.55) | 0 (0, 2.26) | 0 (0, 1.67) | 2.65 (0, 8.47) | 0 (0, 2.38) | 6.16 (0, 10.00) |
|  | 11–13 | 421 | 9.52 (8.12, 10.00) | 5.76 (4.06, 8.16) | 0 (0, 1.19) | 0.37 (0, 3.12) | 3.30 (0, 10.00) | 0 (0, 0.58) | 0 (0, 5.79) |
|  | 14–17 | 323 | 10.00 (8.39, 10.00) | 4.92 (3.76, 6.57) | 0 (0, 1.34) | 0 (0, 2.07) | 4.17 (0, 10.00) | 0 (0, 3.24) | 0 (0, 8.43) |
| Ethnicity | Han | 505 | 9.81 (8.05, 10.00) | 5.94 (4.20, 8.05) | 0 (0, 1.90) | 0 (0, 1.48) | 3.11 (0, 9.16) | 0 (0, 3.51) | 0 (0, 9.79) |
|  | Hani | 109 | 10.00 (8.85, 10.00) | 4.89 (3.90, 6.67) | 0 | 0 (0, 2.37) | 0 (0, 6.80) | 0 (0, 2.39) | 0 (0, 10.00) |
|  | Miao | 106 | 10.00 (8.21, 10.00) | 6.20 (4.58, 7.97) | 0 | 0 (0, 1.62) | 6.40 (0, 10.00) | 0 (0, 2.24) | 0 |
|  | Wa | 104 | 9.14 (7.56, 10.00) | 5.51 (3.32, 7.25) | 1.21 (0, 6.24) | 1.92 (0.37, 4.28) | 8.16 (3.35, 10.00) | 0 (0, 1.70) | 0 (0, 10.00) |
|  | Other | 254 | 10.00(8.37, 10.00) | 5.41(4.18, 7.18) | 0(0, 2.28) | 0.66(0, 2.93) | 2.13(0, 8.71) | 0(0, 0.05) | 0(0, 9.66) |
| Residence | Urban | 245 | 9.50 (8.19, 10.00) | 5.07 (3.63, 6.89) | 0 (0, 5.13) | 1.17 (0, 3.81) | 5.36 (0, 10.00) | 0 (0, 1.30) | 0 (0, 10.00) |
|  | Rural | 833 | 10.00 (8.24, 10.00) | 5.98 (4.31, 7.78) | 0 (0, 0.03) | 0 (0, 1.74) | 2.51 (0, 8.74) | 0 (0, 2.53) | 0 (0, 9.41) |
| Left-behind Status | Left-behind | 244 | 10.00 (8.36, 10.00) | 6.28 (4.63, 8.40) | 0 | 0 (0, 1.30) | 4.45 (0, 8.94) | 0 (0, 1.23) | 0 |
|  | Non-left-behind | 834 | 9.83 (8.22, 10.00) | 5.45 (3.99, 7.35) | 0 (0, 2.34) | 0 (0, 2.88) | 3.30 (0, 10.00) | 0 (0, 2.81) | 0 (0, 10.00) |
| Father’s Education | Elementary or below | 440 | 10.00 (8.38, 10.00) | 5.40 (4.20, 7.54) | 0 | 0 (0, 1.51) | 1.35 (0, 9.16) | 0 (0, 2.98) | 0 (0, 9.90) |
|  | Middle School | 509 | 9.93 (8.33, 10.00) | 6.07 (4.36, 7.76) | 0 (0, 1.18) | 0 (0, 2.15) | 3.60 (0, 10.00) | 0 (0, 2.28) | 0 (0, 9.58) |
|  | College and Above | 129 | 9.06 (7.07, 10.00) | 5.02 (2.98, 6.88) | 3.67 (0, 8.48) | 1.85 (0, 5.30) | 5.34 (1.08, 10.00) | 0 (0, 1.59) | 5.30 (0, 10.00) |
| Mother’s Education | Elementary or below | 496 | 9.91 (8.09, 10.00) | 5.46 (4.12, 7.74) | 0 | 0 (0, 1.78) | 2.70 (0, 9.04) | 0 (0, 2.97) | 0 (0, 6.75) |
|  | Middle School | 450 | 10.00 (8.60, 10.00) | 6.20 (4.50, 7.57) | 0 (0, 1.50) | 0 (0, 1.78) | 3.29 (0, 10.00) | 0 (0, 1.84) | 0 (0, 10.00) |
|  | College and Above | 132 | 9.09 (7.16, 10.00) | 4.63 (3.32, 6.63) | 2.83 (0, 7.90) | 2.06 (0, 5.21) | 5.26 (0.98, 10.00) | 0 (0, 2.69) | 0 (0, 10.00) |
| Household Average Annual Income (10,000 RMB) | <1.5 | 466 | 10.00 (8.37, 10.00) | 6.09 (4.36, 7.61) | 0 | 0 | 3.21 (0, 9.57) | 0 (0, 2.94) | 0 (0, 9.11) |
|  | 1.5–3.5 | 506 | 9.85 (8.05, 10.00) | 5.41 (4.07, 7.53) | 0 (0, 1.72) | 1.02 (0, 2.94) | 3.40 (0, 10.00) | 0 (0, 3.12) | 0 (0, 10.00) |
|  | >3.5 | 106 | 9.42 (7.99, 10.00) | 5.06 (3.16, 8.46) | 2.26 (0, 6.61) | 1.29 (0, 3.51) | 4.00 (0, 9.76) | 0 | 0 (0, 8.28) |
| Overall |  | 1078 | 9.91 (8.24, 10.00) | 5.63 (4.09, 7.59) | 0 (0, 1.74) | 0 (0, 2.37) | 3.48 (0, 9.70) | 0 (0, 2.46) | 0 (0, 9.85) |

| Demographic Indicator | Option | Sample Size | Egg Products | Water Intake | Sugary Drinks | Vitamin A | Dietary Fiber | Food Variety | Daily Energy Intake |
| --- | --- | --- | --- | --- | --- | --- | --- | --- | --- |
| Gender | Male | 497 | 5.52 (0, 8.29) | 4.10 (2.43, 5.50) | 9.33 (7.60, 10.00) | 2.14 (1.33, 3.25) | 3.14 (1.62, 5.77) | 4.00 (3.33, 4.58) | 7.73 (5.17, 9.31) |
|  | Female | 581 | 5.62 (0, 8.96) | 4.92 (2.79, 5.50) | 9.20 (7.33, 10.00) | 2.45 (1.76, 3.89) | 3.22 (1.63, 5.59) | 4.00 (3.50, 5.00) | 8.18 (6.39, 9.71) |
| Age (Years) | 7–10 | 334 | 4.79 (0, 9.20) | 5.50 (2.67, 5.50) | 10.00 (8.13, 10.00) | 3.45 (2.17, 4.38) | 2.40 (1.31, 4.37) | 3.67 (3.33, 4.33) | 8.15 (6.62, 9.73) |
|  | 11–13 | 421 | 6.23 (0, 8.75) | 4.21 (2.12, 5.67) | 9.20 (7.60, 10.00) | 2.22 (1.57, 3.19) | 3.91 (1.87, 6.74) | 4.00 (3.67, 4.67) | 8.70 (7.15, 9.85) |
|  | 14–17 | 323 | 4.97 (0, 8.15) | 4.15 (3.11, 5.41) | 8.40 (6.53, 10.00) | 1.81 (1.22, 2.66) | 3.56 (1.76, 5.67) | 4.33 (3.67, 5.00) | 6.07 (3.74, 8.33) |
| Ethnicity | Han | 505 | 6.20 (0.97, 9.01) | 4.23 (2.78, 5.50) | 10.00 (7.60, 10.00) | 2.44 (1.66, 3.47) | 3.08 (1.68, 5.68) | 4.00 (3.33, 4.42) | 7.95 (5.67, 9.53) |
|  | Hani | 109 | 2.36(0, 7.53) | 4.23(2.68, 5.50) | 10.00(7.77, 10.00) | 2.17(1.22, 4.00) | 1.76(1.26, 4.85) | 3.67(3.33, 4.33) | 7.72(5.19, 9.10) |
|  | Miao | 106 | 6.01(0, 9.17) | 5.00(3.54, 5.75) | 8.92(7.60, 10.00) | 2.06(1.22, 3.09) | 4.19(1.92, 6.36) | 4.00(3.67, 4.67) | 7.65(5.16, 9.54) |
|  | Wa | 104 | 3.63(0, 8.06) | 2.73(2.09, 4.45) | 7.88(4.88, 9.20) | 2.72(1.66, 4.07) | 4.45(2.49, 7.02) | 4.67(4.00, 5.33) | 8.87(7.05, 10.00) |
|  | Other | 254 | 5.00(0, 7.66) | 4.84(2.73, 5.50) | 8.67(6.77, 10.00) | 2.29(1.53, 3.69) | 3.09(1.47, 5.12) | 4.00(3.33, 5.00) | 8.05(6.05, 9.66) |
| Residence | Urban | 245 | 1.57(0, 7.78) | 3.08(2.09, 5.50) | 8.27(5.33, 10.00) | 2.31(1.29, 3.96) | 4.16(2.24, 6.20) | 4.33(3.67, 5.33) | 8.76(7.32, 10.00) |
|  | Rural | 833 | 5.94(0.67, 8.84) | 4.53(3.00, 5.50) | 9.47(7.60, 10.00) | 2.33(1.64, 3.46) | 3.04(1.55, 5.47) | 4.00(3.33, 4.33) | 7.72(5.48, 9.36) |
| Left-behind Status | Left-behind | 244 | 6.26(0.07, 8.74) | 5.00(2.46, 6.13) | 10.00(7.87, 10.00) | 2.31(1.60, 3.20) | 3.63(1.85, 5.67) | 4.00(3.67, 4.33) | 8.10(6.47, 9.51) |
|  | Non-left-behind | 834 | 5.09(0, 8.57) | 4.23(2.73, 5.50) | 9.20(7.20, 10.00) | 2.34(1.55, 3.70) | 3.08(1.54, 5.70) | 4.00(3.33, 5.00) | 7.92(5.69, 9.61) |
| Father’s Education | Elementary or below | 440 | 5.63(0, 8.30) | 4.50(2.67, 5.50) | 9.20(7.60, 10.00) | 2.06(1.42, 3.05) | 2.78(1.47, 5.39) | 4.00(3.33, 4.67) | 7.76(5.33, 9.27) |
|  | Middle School | 509 | 5.74(0, 9.15) | 4.42(3.03, 5.50) | 10.00(7.60, 10.00) | 2.56(1.70, 3.73) | 3.33(1.68, 5.94) | 4.00(3.33, 4.67) | 8.10(5.90, 9.71) |
|  | College and Above | 129 | 3.84(0, 7.83) | 3.03(1.97, 5.36) | 7.33(4.93, 9.33) | 2.93(1.63, 5.07) | 3.94(2.37, 5.72) | 5.33(4.00, 5.67) | 8.30(6.90, 9.78) |
| Mother’s Education | Elementary or below | 496 | 5.91(0.94, 8.56) | 4.23(2.59, 5.53) | 9.20(7.60, 10.00) | 2.06(1.46, 3.08) | 3.04(1.60, 5.57) | 4.00(3.33, 4.67) | 7.86(5.50, 9.42) |
|  | Middle School | 450 | 4.63(0, 8.95) | 4.59(3.07, 5.50) | 10.00(7.60, 10.00) | 2.61(1.69, 4.09) | 3.14(1.49, 5.69) | 4.00(3.33, 4.67) | 8.01(5.75, 9.63) |
|  | College and Above | 132 | 3.87(0, 8.16) | 3.22(1.97, 5.50) | 8.27(5.60, 9.93) | 2.94(1.94, 4.30) | 3.92(2.37, 5.88) | 5.00(4.00, 5.67) | 8.57(6.91, 9.90) |
| Household Average Annual Income (10,000 RMB) | <1.5 | 466 | 5.68(0, 8.88) | 4.23(2.50, 5.50) | 10.00(7.60, 10.00) | 2.25(1.50, 3.43) | 3.25(1.58, 5.62) | 4.00(3.33, 4.33) | 7.78(5.48, 9.41) |
|  | 1.5–3.5 | 506 | 5.69(0, 8.58) | 4.23(2.73, 5.50) | 9.20(7.33, 10.00) | 2.30(1.67, 3.49) | 3.17(1.60, 5.87) | 4.00(3.33, 4.67) | 8.10(5.95, 9.71) |
|  | >3.5 | 106 | 3.86(0, 7.88) | 4.71(2.80, 5.83) | 7.80(5.60, 9.82) | 2.77(1.62, 4.42) | 3.06(2.24, 5.52) | 4.58(3.92, 5.33) | 8.27(6.90, 9.68) |
| Overall |  | 1078 | 5.58(0, 8.58) | 4.23(2.67, 5.50) | 9.20(7.38, 10.00) | 2.33(1.56, 3.53) | 3.19(1.63, 5.67) | 4.00(3.33, 4.67) | 7.99(5.87, 9.60) |

Table 10. Comparion of total dietary index scores of children and adolescents aged 7－17 among different groups in Yunnan Province [M (P25, P75)]

| Demographic Indicator | Options | Sample Size | Total dietary score | Z/H value | P value |
| --- | --- | --- | --- | --- | --- |
| Gender | Male | 497 | 59.32(52.60, 69.72) | -5.16 | <0.01 |
|  | Female | 581 | 64.20(56.12, 72.56) |  |  |
| Age (Years) | 7–10 | 334 | 63.46(56.19, 72.63) | 32.23 | <0.01 |
|  | 11–13 | 421 | 65.35(54.29, 72.03) |  |  |
|  | 14–17 | 323 | 59.07(51.95, 68.30) |  |  |
| Ethnicity | Han | 505 | 63.71(55.21, 71.53) | 27.51 | <0.01 |
|  | Hani | 109 | 57.84(53.44, 64.55) |  |  |
|  | Miao | 106 | 62.29(53.46, 72.17) |  |  |
|  | Wa | 104 | 68.78(55.63, 76.86) |  |  |
|  | Other | 254 | 60.21(53.18, 70.52) |  |  |
| Residence | Left-behind | 245 | 65.30(54.84, 73.62) | -2.11 | 0.04 |
|  | Non-left-behind | 833 | 62.17(54.31, 70.70) |  |  |
| Left-behind Status | Urban | 244 | 62.65(55.15, 70.26) | -0.23 | 0.82 |
|  | Rural | 834 | 62.61(54.28, 71.65) |  |  |
| Father’s Education | Elementary or below | 440 | 59.44(53.72, 69.07) | 27.68 | <0.01 |
|  | Middle School | 509 | 63.48(55.34, 71.96) |  |  |
|  | College and Above | 129 | 68.72(55.57, 78.70) |  |  |
| Mother’s Education | Elementary or below | 496 | 61.00(53.06, 69.87) | 22.58 | <0.01 |
|  | Middle School | 450 | 63.01(55.50, 71.64) |  |  |
|  | College and Above | 132 | 68.65(55.62, 78.63) |  |  |
| Household Average Annual Income (10,000 RMB) | <1.5 | 466 | 61.48(54.17, 70.18) | 4.66 | 0.10 |
|  | 1.5–3.5 | 506 | 63.41(55.20, 71.70) |  |  |
|  | >3.5 | 106 | 64.48(53.45, 73.43) |  |  |

1. Li Y, Yang L, Jiang M, Huang Z, Wu H, Lai S. Evaluation of Dietary Quality of School-Age Children in Fujian Province. Acta Nutr Sin. (2022) 44(5):436–41. doi:10.13325/j.cnki.acta.nutr.sin.2022.05.002

2. Qiao T, Duan R, Cheng G. Revision of the Chinese Children Dietary Index. Acta Nutr Sin. (2019) 41(2):105–9. doi:10.13325/j.cnki.acta.nutr.sin.2019.02.002

3. Ran F, Zi F, Lu X, Mao Y, Mu D, Zhang S, et al. Diet Quality of Primary and Secondary School Students in Yunnan Province. Chin J Sch Health. (2023) 44(7):985–90. doi:10.16835/j.cnki.1000-9817.2023.07.006
